# Ductile, High-Lignin-Content Thermoset Films and Coatings

**DOI:** 10.1021/acssuschemeng.3c03030

**Published:** 2023-11-09

**Authors:** Alice Boarino, Justine Charmillot, Monique Bernardes Figueirêdo, Thanh T. H. Le, Nicola Carrara, Harm-Anton Klok

**Affiliations:** † Institut des Matériaux and Institut des Sciences et Ingénierie Chimiques, Laboratoire des Polymères, École Polytechnique Fédérale de Lausanne (EPFL), Station 12, CH-1015 Lausanne, Switzerland; ‡ Bloom Biorenewables, Route de l’Ancienne Papeterie 106, CH-1723 Marly, Switzerland

**Keywords:** aldehyde-assisted fractionation, epoxy cross-linkers, food packaging, UV barrier, antioxidant activity

## Abstract

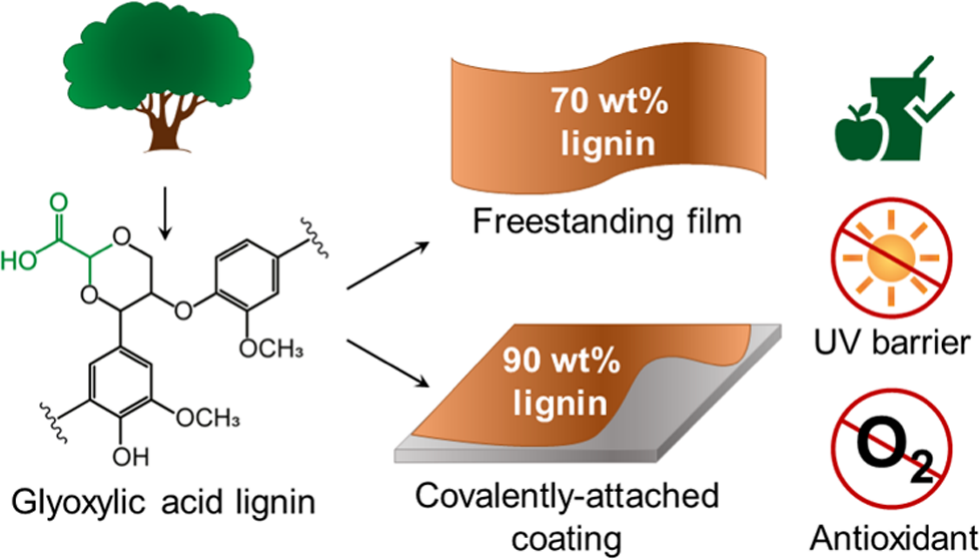

In the context of transitioning toward a more sustainable use of natural resources, the application of lignin to substitute commonly utilized petroleum-based plastics can play a key role. Although lignin is highly available at low cost and presents interesting properties, such as antioxidant and UV barrier activities, its application is limited by its low reactivity, which is a consequence of harsh conditions normally used to extract lignin from biomass. In this work, the use of glyoxylic acid lignin (GA lignin), rich in carboxylic acid groups and hence highly reactive toward epoxy cross-linkers, is presented. GA lignin, which is directly extracted from biomass via a one-step aldehyde-assisted fractionation process, allowed the preparation of thermoset films and coatings via a simple reaction with sustainable poly(ethylene glycol) diglycidyl ether and glycerol diglycidyl ether cross-linkers. This allows one to prepare freestanding films containing up to 70 wt % lignin with tunable mechanical properties and covalently surface-attached coatings containing up to 90 wt % lignin with high solvent resistance. Both films and coatings display antioxidant properties and combine excellent UV barrier activity with high visible transparency, which is attractive for applications in sustainable food packaging.

## Abstract 2

Glyoxylic acid lignin, a biomass-derived polymer easily available in high quantity, can be used to prepare thermoset films and coatings with properties that make them attractive for food packaging applications.

## Introduction

The production of chemicals and materials from renewable sources to substitute the currently used petroleum derivatives is necessary to reduce our dependence on fossil fuels and their environmental impact. One field where this transition can have a great impact is the market of polymer materials, which are ubiquitous and produced in huge quantities (more than 350 million tons per year).^1−3^ A promising candidate for the substitution of oil-derived polymer materials is lignin, the most abundant natural source of aromatics on our planet. Lignin constitutes 15–35% of lignocellulosic biomass and is produced in large amounts as a side stream from the paper and bioethanol industry.^4,5^ About 98% of the 100 million tons of lignin produced every year is burned as a low-value energy source, so its valorization is very attractive both from the point of view of sustainability as well as from the perspective of transitioning toward a circular polymer economy.^6−8^ Besides its wide abundance and low cost, lignin also possesses attractive characteristics, such as antioxidant and UV barrier activities. These properties are due to the high phenol content of lignin. These phenol groups can act as radical scavengers to inhibit oxidation reactions,^9,10^ as well as chromophores that can absorb UV light.^11,12^ Thanks to these properties, lignin has been widely used in food packaging films and coatings, where it can help to preserve the food and prolong its shelf life.^13^

A fundamental challenge in the use of lignin is that it is very brittle. Hence, lignin is often combined with other polymers to generate blends or composite materials.^14,15^ The formation of films and coatings with high lignin content is challenging, as the brittleness and heterogeneous chemical structure of lignin typically lead to poor mechanical properties at high lignin contents (a maximum of 20–30 wt % lignin can be normally reached, without chemical modification).^14^ Another major problem with the application of lignin is its dark brown color and opacity in the visible range, which strongly limits its use in packaging films and coatings. In order to maintain sufficient transparency, a very low lignin content must generally be adopted (<10 wt %).^16^

Chemical cross-linking of lignin provides access to high-lignin-content thermoset materials.^17^ One way to accomplish this is by direct cross-linking of unmodified lignin. This strategy was explored by Xu et al., who prepared a thermoset by reacting an unmodified Kraft lignin with citric acid and poly(ethylene glycol).^18^ A challenge with the use of conventional extracted lignin, such as Kraft lignin, is that their extraction generally involves high temperature and strongly acidic or alkaline conditions,^19^ which are accompanied by irreversible and uncontrolled condensation reactions that reduce the amount of available hydroxyl groups and can result in the formation of stable carbon–carbon bonds between the lignin units. Therefore, lignin extracted from biomass is often further fractionated and chemically modified to introduce new functional groups prior to the formation of thermoset materials. Gioia et al. reported the preparation of thermoset films starting from a Kraft lignin that was first fractionated by sequential solvent extraction, then modified with epichlorohydrin, and finally reacted with poly(propylene oxide) diamine cross-linkers.^20,21^ In the two cited papers, thermosets containing 41 and 66 wt % modified lignin were obtained. In another example, Ribca et al. prepared a thermoset film using a Kraft lignin that was fractionated, subsequently modified by reaction with allyl chloride, and then cross-linked using a multifunctional thiol-based cross-linker.^22^ In this case, a thermoset with 68 wt %-modified lignin content was achieved. Hao et al. produced thermoset coatings containing 47 wt % Kraft lignin, which was first esterified with 4-methylcyclohexane-1,2-dicarboxylic anhydride in pyridine and then cross-linked with poly(ethylene glycol) diglycidyl ether (PEGDE).^23^

This paper describes an alternative approach toward the preparation of ductile, high-lignin-content films and coatings, which requires neither fractionation nor postfunctionalization of lignin prior to the formation of the final thermoset. The method reported in this paper takes advantage of the use of aldehyde derivatives during the lignin extraction process, which allows for a one-step extraction and isolation of functional lignins without the need for a separate postmodification step. The aldehyde-assisted fractionation (AAF) process that was used to isolate the lignin explored in this paper uses an aldehyde as a protection agent during the lignin extraction process.^24^ The aldehyde reacts with the hydroxyl groups from the prevalent β-O-4 lignin linkage, creating an acetal, thus preventing lignin condensation (the AAF mechanism is represented in the Supporting Information, Scheme S1). The choice of aldehyde determines the structure of the extracted lignin, and consequently its solubility and properties.^25^ The AAF technology enables the simultaneous isolation of high-quality lignin from biomass and its controlled functionalization.^26^ To extract the lignin utilized in this work, glyoxylic acid (GA) has been used as the protection group during AAF. The use of glyoxylic acid not only allows the preservation of hydroxyl functional groups and prevention of carbon–carbon bond formation during the extraction process but also simultaneously introduces carboxylic acid functional groups,^27^ which are versatile reactive handles, for example, for further modification reactions or for the preparation of thermoset materials.

In this study, two epoxy cross-linkers, poly(ethylene glycol) diglycidyl ether (PEGDE) and glycerol diglycidyl ether (GDE), were blended with GA lignin to produce films and coatings with high lignin content, which are potentially 100% biobased. PEGDE is derived from poly(ethylene glycol), recognized as biocompatible and biodegradable,^28^ while GDE is a derivative of glycerol, a byproduct of biodiesel production.^29^ While epoxide chemistry has been previously explored for the cross-linking of lignin,^17,18,20,21^ the high carboxylic acid group content of the GA lignin obtained via the AAF process facilitates the reaction with epoxide cross-linkers. The chemistry of the carboxylic acid–epoxide reaction is well-established and is taken advantage of in the curing of epoxy resins.^30^ This study leverages the very high functional group content of GA lignin obtained via the AAF process with this well-known reaction to provide a simple path toward ductile, high-lignin-content thermoset films and coatings. The first part of this paper reports on the reaction between GA lignin and the two bis-epoxide cross-linkers in model experiments in the solution. Freestanding films with lignin contents of 50, 60, and 70 wt % and tunable mechanical properties were then produced by blending and cross-linking of GA lignin and the bis-epoxide cross-linkers. Blending and cross-linking GA lignin with bis-epoxides not only provides access to freestanding films but also allows the preparation of surface coatings that are robustly and covalently attached to silicon wafer substrates. Coatings with 50, 70, or 90 wt % GA lignin were prepared, which were covalently attached to the substrate and displayed excellent stability against organic solvents. The freestanding films and coatings reported in this paper have been obtained via straightforward and sustainable processes, which do not involve the use of any catalyst or additive and only require heating of the GA lignin/cross-linker formulation at 120 °C. Both the freestanding films and the surface-attached coatings combine excellent UV barrier properties with transparency in the visible light region. The antioxidant activity of lignin was also maintained in the prepared films and coatings, making them promising materials for applications in food packaging and preservation.

## Experimental Section

A detailed description of the materials and methods utilized in this study is reported in the Supporting Information.

## Procedures

### Model Reactions between GA Lignin and Bis-epoxides in Solution

0.5 g of GA lignin (1.4 mmol of aliphatic hydroxyl groups, 0.5 mmol of phenol groups, and 0.4 mmol of carboxylic acid groups) was dissolved in 10 mL of dioxane. Then, 0.5 g of PEGDE (2 mmol epoxy groups) or 0.5 g of GDE (5 mmol of epoxy groups) was added to the solution, and the mixture was heated to 100 °C for 5 h under stirring. The solution was then allowed to cool to room temperature, and the solvent subsequently evaporated under reduced pressure to obtain a viscous liquid. The mixture was then transferred into a dialysis membrane with a molecular weight cutoff of 1 kDa (dialysis membrane spectra/PorRTM 7 pore size 1000, 38 mm, Carl Roth GmbH & Co.) and dialyzed against water for 3 days. The remaining solution was finally freeze-dried.

### Preparation of Freestanding Lignin Films

First, 30 g of GA lignin was dissolved in 100 mL of dioxane. After that, an appropriate amount of PEGDE or GDE (depending on the desired content in the final film, as summarized in the Supporting Information, Table S1) was added to 15 mL of the GA lignin solution, and the mixture was stirred for 30 min at 300 rpm. The mixture was then poured into a Teflon (PTFE) dish (10.2 cm diameter) and left in a fume hood for 48 h to evaporate the solvent. After that, the film was fully dried and cured in an oven using the following temperature ramp: 30 min at 80 °C, 30 min at 100 °C, and 3 h at 120 °C. The films had an average thickness of 0.3–0.4 mm, as measured with a micrometer in at least three different regions of the film.

### Preparation of Freestanding Poly(lactic acid) (PLA) Films

Food packaging grade PLA films were prepared via solution casting. PLA was first dried overnight at 40 °C in a vacuum oven, dissolved in chloroform to obtain a 10 wt % solution, and then cast onto a Teflon Petri dish. The film was left drying for 24 h at room temperature and then for 72 h in a vacuum oven at 50 °C. It was finally removed from the mold and tested. The films had an average thickness of 0.3–0.4 mm, as measured with a micrometer in at least three different regions of the film.

### Determination of the Sol Fraction of the Freestanding Films

A precisely weighted amount of each film sample (typically between 80 and 100 mg) was immersed in 1.3 mL of dioxane, vortexed, and then left submerged for 18 h. The samples were then recovered, dried under vacuum (<20 mbar) at 60 °C for 6.5 h, and weighed again. The sol fraction was calculated using the following equation considering the ratio between the final weight of the washed and dried sample (*W*_f_) and the initial sample weight (*W*_i_):
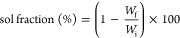


### Preparation of Surface-Attached Lignin Coatings

Two types of substrates were used for the preparation of the coatings: silicon wafers (10 × 8 mm size) were used for the DPPH test and scanning electron microscopy (SEM) analysis and fused silica wafers (circular with a 25 mm diameter) were used to prepare samples to study optical properties. Before applying the coating, the surfaces were cleaned by ultrasonication in ethanol, water, and acetone for 8 min each. The substrates were then placed in a Femto Oxygen Plasma system (200 W, Diener Electronic) under 5 mL/min oxygen flow for 15 min for surface activation. Coatings were prepared from 200 mg/mL solutions of lignin in dioxane. An appropriate amount of either PEGDE or GDE (depending on the desired content in the final coating, as summarized in the Supporting Information, Table S2) was added to the lignin solution in order to obtain the desired weight ratio of lignin/bis-epoxide. After mixing, the solution was pipetted on the clean activated substrate (40 μL was needed for 10 mm × 8 mm rectangular wafers and 200 μL for circular substrates with *d* = 25 mm). The coated substrates were then placed under a vacuum overnight at room temperature to allow the evaporation of the solvent. Finally, the surfaces were heated at 120 °C for 3 h to complete the cross-linking. Coatings with average thicknesses between 0.13 and 0.16 mm, as measured with a micrometer in at least three different regions of the surface, were obtained.

## Results and Discussion

### Model Reactions between GA Lignin and Bis-epoxides in Solution

**Scheme 1:**
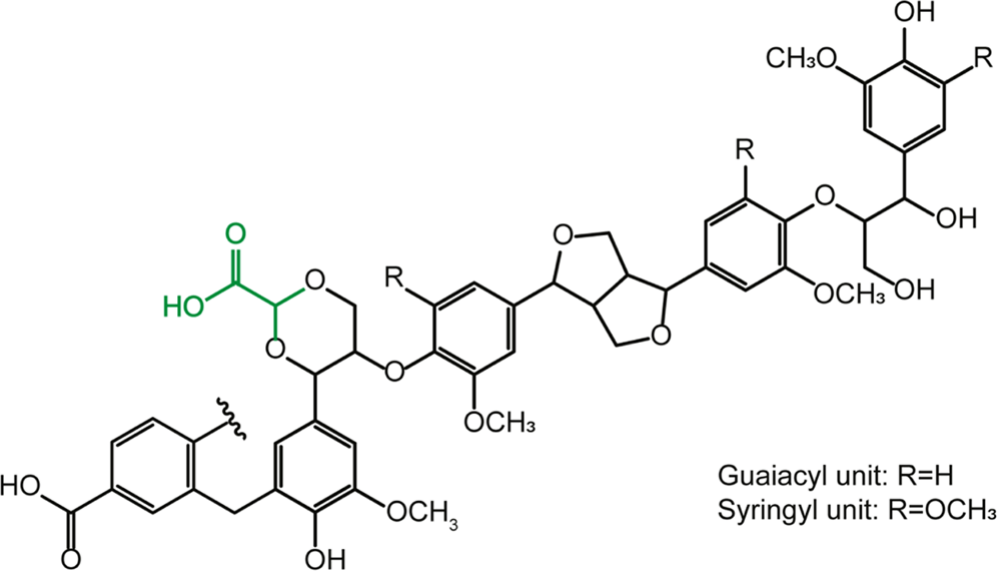
Schematic Representation of the Structure of GA Lignin

Prior to exploring the use of GA lignin to prepare freestanding films and covalently attached surface coatings, its reactivity toward the two bis-epoxide cross-linkers used in this study [poly(ethylene glycol) diglycidyl ether (PEGDE) and glycerol diglycidyl ether (GDE)] was investigated in model experiments in solution. The structure of GA lignin used in this study, which was confirmed by HSQC-NMR experiments (Supporting Information, Figure S1), is illustrated in Scheme 1. To determine the contents of various hydroxyl and carboxylic acid groups, GA lignin was analyzed by ^31^P NMR spectroscopy. The results of these experiments, which are presented in Supporting Information Figures S2 and S3, and in Supporting Information Table S3, indicate that the GA lignin used in this study contains 2.83 mmol/g aliphatic hydroxyl groups, 0.76 mmol/g syringyl phenol groups, 0.37 mmol/g guaiacyl phenol groups, and 0.82 mmol/g carboxylic acid groups. This carboxylic acid group content is significantly higher as compared to that of other lignins reported in the literature, which is typically 0.02–0.29 mmol/g.^31^

**Figure 1:**
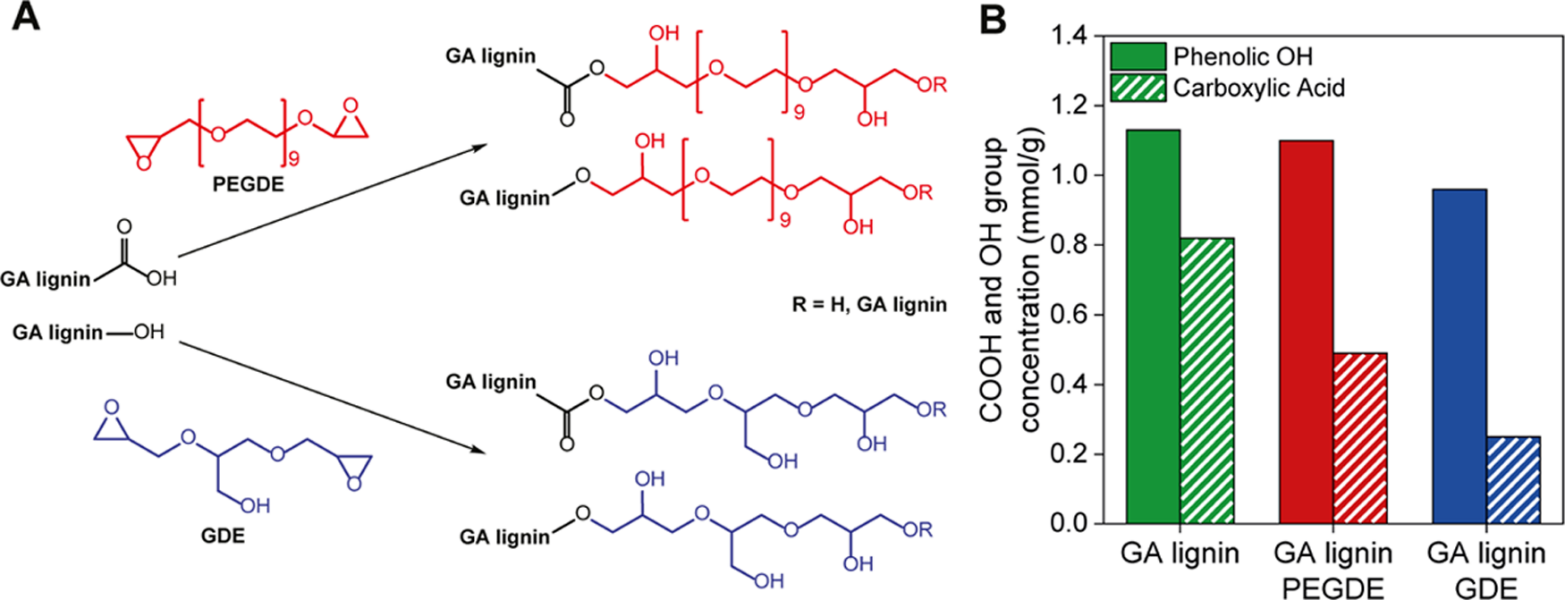
(A) Reaction of GA lignin with PEGDE and GDE. (B) Carboxylic acid and phenol group content in GA lignin before and after the reaction with PEGDE and GDE (50 wt % of GA lignin and 50 wt % of cross-linker), as determined by ^31^P NMR spectroscopy.

To investigate the reactivity of GA lignin toward the bis-epoxide cross-linkers used in this study, 50 mg/mL dioxane solutions of GA lignin containing equivalent weights of GDE or PEGDE were heated at 100 °C for 5 h (Figure 1A). The reaction products were purified by dialysis to remove unreacted epoxide, freeze-dried, and subsequently analyzed by NMR and Fourier transform infrared (FTIR) spectroscopy. Comparison of the HSQC-NMR spectra of the GA lignin before and after the reaction with PEGDE and GDE indicated that the reaction did not significantly affect the lignin structure (Supporting Information, Figures S4 and S5). Most notably, the HSQC-NMR spectra of the reaction products feature the characteristic signal of the acetal group at δH/δC = 5/100 ppm (highlighted as GA_1_ in the HSQC-NMR spectrum), which is due to the functionalization of lignin with glyoxylic acid during the AAF extraction process. The epoxide groups of the PEGDE and GDE cross-linkers can potentially react with the carboxylic acid and aliphatic hydroxyl and phenol groups of GA lignin.^30^ To identify the nature of the functional groups in GA lignin that participates in the reaction with PEGDE and GDE, the PEGDE- and GDE-modified GA lignin samples were analyzed by ^31^P NMR spectroscopy (Supporting Information, Figures S2 and S3). The results of these analyses, which are presented in Figure 1B and summarized in the Supporting Information, Table S3, indicated that the reaction with PEGDE and GDE does not significantly involve the lignin phenol groups. While ^31^P NMR spectroscopy reveals a decrease of the carboxylic acid content from 0.82 to 0.49 and 0.25 mmol/g after reacting with PEGDE and GDE, respectively, the syringyl and guaiacyl phenol group content is only slightly reduced from 1.13 mmol/g for GA lignin to 1.10 mmol/g from the PEGDE-modified material and 0.96 mmol/g after the reaction with GDE. The larger reduction in carboxylic acid group content in the GDE-modified GA lignin was expected because GDE has a lower molecular weight than PEGDE; hence, the same weight of the cross-linker corresponds to a larger number of moles of GDE, consequently reacting with a higher amount of GA lignin carboxylic acid groups. The insignificant, respectively, minor reduction in the phenol group content is attractive since these groups contribute to the antioxidant and UV-absorbing activity of lignin.^11,12^ Due to the ring opening of the epoxy groups, a significant increase of the aliphatic hydroxyl group content was observed from 2.83 mmol/g in the GA lignin to 6.89 and 14.14 mmol/g after the reaction with PEGDE and GDE, respectively.

### Freestanding GA Lignin Films

After investigating the reaction between GA lignin and PEGDE and GDE in model experiments, freestanding, cross-linked films were prepared by thermal curing of mixtures of GA lignin and the two bis-epoxides. These films were prepared by dissolving GA lignin and the appropriate cross-linker in dioxane, followed by casting the polymer solution into a PTFE mold. After evaporation of the solvent, the films were thermally cured at temperatures up to 120 °C. This procedure proved to be efficient and reproducible and avoided the use of catalysts or harsh conditions. The temperatures that are used to produce the films are lower than those that are typically applied to process, for example, poly(lactic acid) food packaging films.^32,33^ Cross-linked GA lignin films were prepared from formulations that contained 50, 60, and 70 wt % of the lignin component (and thus, 50, 40, and 30 wt % of bis-epoxide). This process afforded round, cross-linked lignin films with a size of 12.5 cm^2^ and a thickness of 0.3–0.4 mm (Supporting Information, Table S4). Supporting Information Figures S6 and S7 present optical micrographs of specimen cut from the different films, both in the unstrained and in the bent state, which highlights the flexibility of the materials.

**Figure 2:**
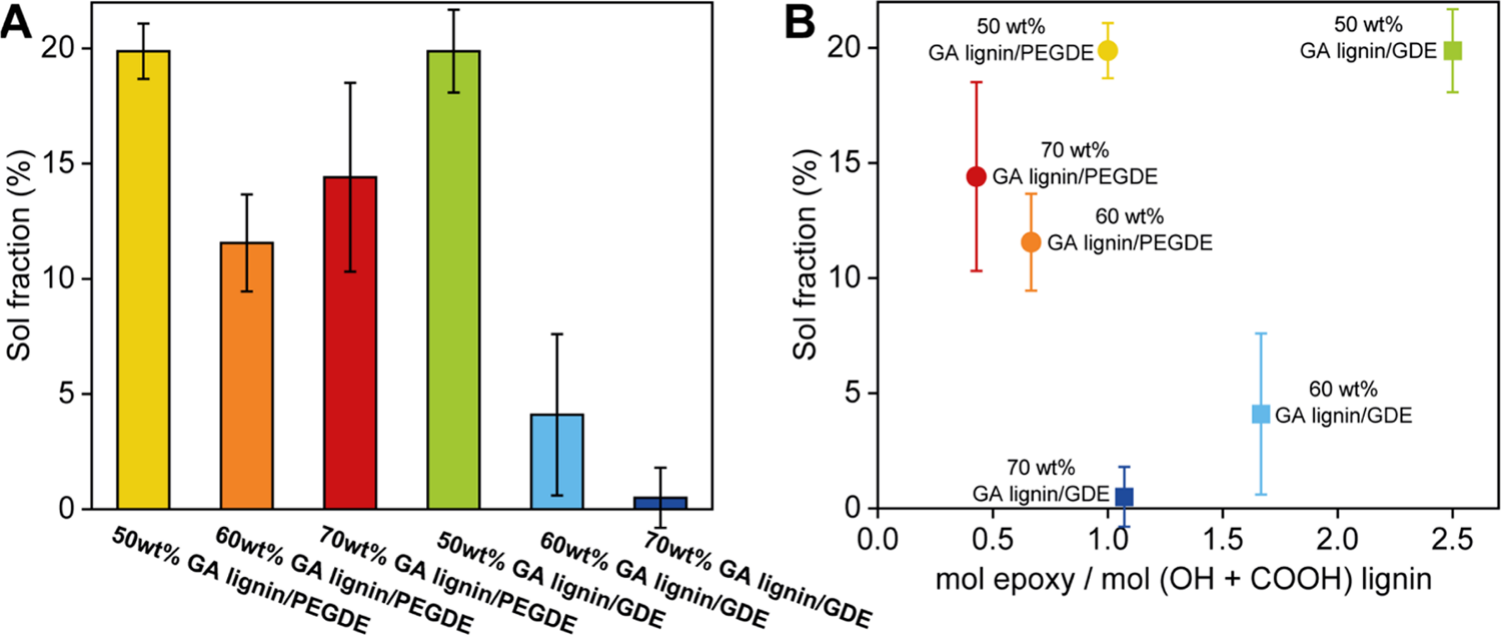
(A) Sol fractions of PEGDE and GDE cross-linked GA lignin films containing 50, 60, or 70 wt % lignin. (B) Sol fraction of PEGDE and GDE cross-linked GA lignin films plotted against the molar ratio of cross-linker epoxy groups and functional groups in GA lignin.

To evaluate the curing process, we determined the sol fraction of the GA lignin films by extracting the soluble components with dioxane. Figure 2A plots the sol fractions for each of the GA lignin films that were prepared. In Figure 2B, the sol fraction of each of the films is presented as a function of the molar ratio of epoxide groups to lignin functional groups (aliphatic hydroxyl, phenol, and carboxylic acid groups) that were used in the curing reaction. The data in Figure 2A show that the sol fraction of the GA lignin/PEGDE films is 10–20% and does not significantly change upon varying the relative amounts of GA lignin and PEGDE. For the GDE cross-linked GA lignin films, in contrast, the sol fraction decreases from 20 to 0.5% when the amount of GDE is decreased from 50 to 30 wt %, i.e., upon decreasing the molar ratio of epoxide groups to lignin hydroxyl and carboxylic acid groups. As illustrated in Figure 2B, the measured sol fractions correlate with the molar ratio of epoxide to lignin hydroxyl and carboxylic acid groups that were used to produce the GA lignin/GDE films. GA lignin/GDE films containing 70 wt % GA lignin are obtained from a formulation that contains essentially stoichiometric quantities of epoxide and GA lignin hydroxyl and alcohol groups, resulting in only a negligible sol fraction of 0.5 wt %. GA lignin/GDE films prepared with 50 and 60 wt % GA lignin are obtained from formulations that present a molar excess of epoxide. The increased sol fractions measured for these films reflect the excess unreacted epoxide cross-linker. The sol fractions measured for the GA lignin/PEGDE films prepared with 50 wt % PEGDE are significantly higher as compared to those of GA lignin/GDE films obtained using 70 wt % GA lignin, even though both are produced from formulations that contain equimolar quantities of epoxide and GA lignin hydroxyl and carboxylic acid groups. This difference suggests that PEGDE is a less efficient cross-linker as compared to GDE, and the sol fraction measured for the films prepared with 50 wt % PEGDE is likely due to both unreacted GA lignin and unreacted PEGDE. Increasing the GA lignin content in the GA lignin/PEGDE films from 50 wt % to 60 and 70 wt % results in an excess of lignin hydroxyl and carboxylic acid groups as compared to epoxide groups. For these last high-lignin-content GA lignin/PEGDE films, the sol fractions, which are not statistically different from each other, therefore likely represent predominantly excess unreacted GA lignin.

To further investigate the curing process, the films were analyzed by FTIR spectroscopy. The spectra, which are included in Supporting Information Figure S8, present the typical lignin peaks at 3400 cm^–1^ (O–H stretching) and 1730 cm^–1^ (C=O stretching). The spectra also show signals belonging to the cross-linker at 2860 cm^–1^ (C–H stretching) and 1090 cm^–1^ (C–H bending), which increase in intensity with an increasing amount of bis-epoxide cross-linker in the film. The FTIR spectra of the films prepared with 50 wt % GA lignin and 50 wt % GDE reveal a very small epoxide band at 900 cm^–1^, indicating the presence of a minor fraction of unreacted epoxide groups.

SEM analysis of the bis-epoxide cross-linked GA lignin films revealed uniform smooth surface morphologies, even for films with the highest lignin content (Supporting Information, Figure S9A). In contrast, when films were prepared using the same protocol but with soda lignin instead of GA lignin, SEM analysis showed surface morphologies that are rough and contain cracks (Supporting Information, Figure S9B). Higher magnification SEM images of the cross-linked GA lignin and soda lignin films are presented in Supporting Information Figure S10.

The thermal properties of the cross-linked GA lignin films were analyzed by DSC and TGA (Supporting Information Table S5, Figures S11 and S12). The *T*_g_ of the GA lignin/PEGDE films increases from −23 to 1 and 64 °C by increasing the GA lignin content from 50 to 60 and 70 wt %. For the GDE cross-linked GA lignin films, the *T*_g_ increases from −5 to 17 and 63 °C upon increasing the lignin content from 50 to 60 and 70 wt %. This was expected because the *T*_g_ of pure GA lignin is 76.5 °C, while the *T*_g_ of the cross-linkers is −70 and −90 °C for PEG_500_^34^ and glycerol,^35^ respectively. Films containing 70 wt % lignin have almost the same *T*_g_, independently from the used cross-linker. When a higher content of cross-linker was used (50 and 60 wt % lignin), the cross-linker type had an influence on the *T*_g_. GA lignin/GDE films have higher *T*_g_ values than GA lignin/PEGDE films. Overall, the obtained *T*_g_ values are in the same range as those of commercial polymers that are commonly used for food packaging, such as poly(ethylene terephthalate) (PET), poly(lactic acid) (PLA), and polypropylene (PP) (Supporting Information Figure S13A). To characterize the thermal stability of the films, the initial thermal degradation temperature (*T*_5%_), i.e., the temperature at which the mass of the sample is 5% lower than its mass at 50 °C, was determined by TGA (Supporting Information, Table S5). The *T*_5%_ increases with decreasing GA lignin content and increasing cross-linker content: 236, 205, and 193 °C for 50, 60, and 70 wt % GA lignin/PEGDE and 249, 202, and 181 °C for 50, 60, and 70 wt % GA lignin/GDE films, respectively. Overall, the recorded *T*_5%_ values are slightly lower than those of commercial polymers, but the films are thermally stable and could thus be used, for example, as food packaging items until the reasonably high temperatures of ∼180 °C (Supporting Information, Figure S13B). The absence of any mass loss in the TGA curves presented in Supporting Information Figure S12 also indicates that the curing and drying processes that used to prepare the films were effective in removing the dioxane solvent.

**Figure 3:**
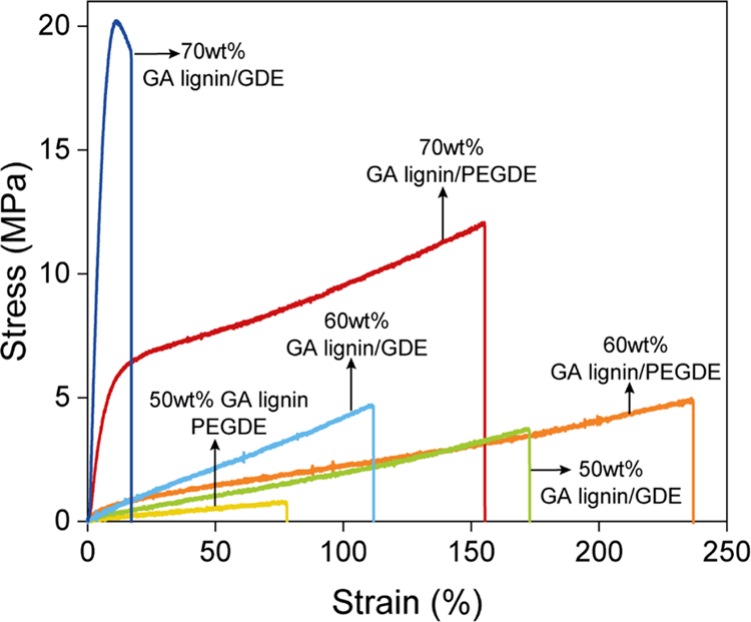
Stress–strain curves of GA lignin/PEGDE and GA lignin/GDE films containing 50, 60, or 70 wt % lignin.

The mechanical properties of the cross-linked GA lignin films were evaluated using tensile testing experiments. Figure 3 presents the stress–strain curves obtained for the different samples. The tensile strength, elongation at break, Young’s modulus, and toughness of the samples are reported in Supporting Information Table S4. For the films made with GDE, increasing the GA lignin content results in an increase in tensile strength (3.5, 4.4, and 16.9 MPa for 50, 60, and 70 wt % GA lignin/GDE, respectively) and a decrease of elongation at break (162, 122, and 11.4% for 50, 60, and 70 wt % GA lignin/GDE, respectively). Except for the 70 wt % GA lignin/GDE film, which shows brittle behavior, all the other cross-linked GA lignin films are ductile. The toughness of the GA lignin/GDE films does not change significantly with different lignin contents (2.45, 2.68, and 2.59 MJ/m^3^ for 50, 60, and 70 wt % GA lignin/GDE, respectively). For the films made with PEGDE, the tensile strength shows a similar trend, increasing with GA lignin content (1.5, 4.5, and 11.8 MPa for 50, 60, and 70 wt % GA lignin/PEGDE, respectively), while the elongation at break follows a different trend with the 60 wt % GA lignin/PEGDE presenting a higher value (235%) than 50 wt % GA lignin/PEGDE (114%) and 70 wt % GA lignin/PEGDE (149%). The correlation between the composition of the cross-linked GA lignin films and their mechanical properties is complex and the result of an intricate interplay of several variables. On the one hand, the PEGDE and GDE content determines the cross-linking density and thereby mechanical properties of the films. On the other hand, blending the soft and flexible ethylene-glycol-based cross-linkers (with *T*_g_ values of −70 and −90 °C) with the higher *T*_g_ GA lignin also impacts mechanical properties. Supporting Information Figure S14 presents the tensile strength and elongation at break of the GA lignin films and compares them with those of various commercial polymers. The GA lignin films present higher elongation at break than polystyrene (PS) and poly(lactic acid) (PLA), with values comparable to poly(propylene) (PP) and poly(ethylene terephthalate) (PET). Their tensile strength is in the same range as polybutadiene rubber (PBR), nitrile butadiene rubber (NBR), and low-density polyethylene (LDPE). When comparing the data for the different polymers summarized in Supporting Information Figure S14, it is important to keep in mind that the mechanical properties of polymer films are strongly influenced by the sample preparation method (for example, solution casting, melt extrusion, or injection molding), sample thickness, and testing conditions.

**Figure 4:**
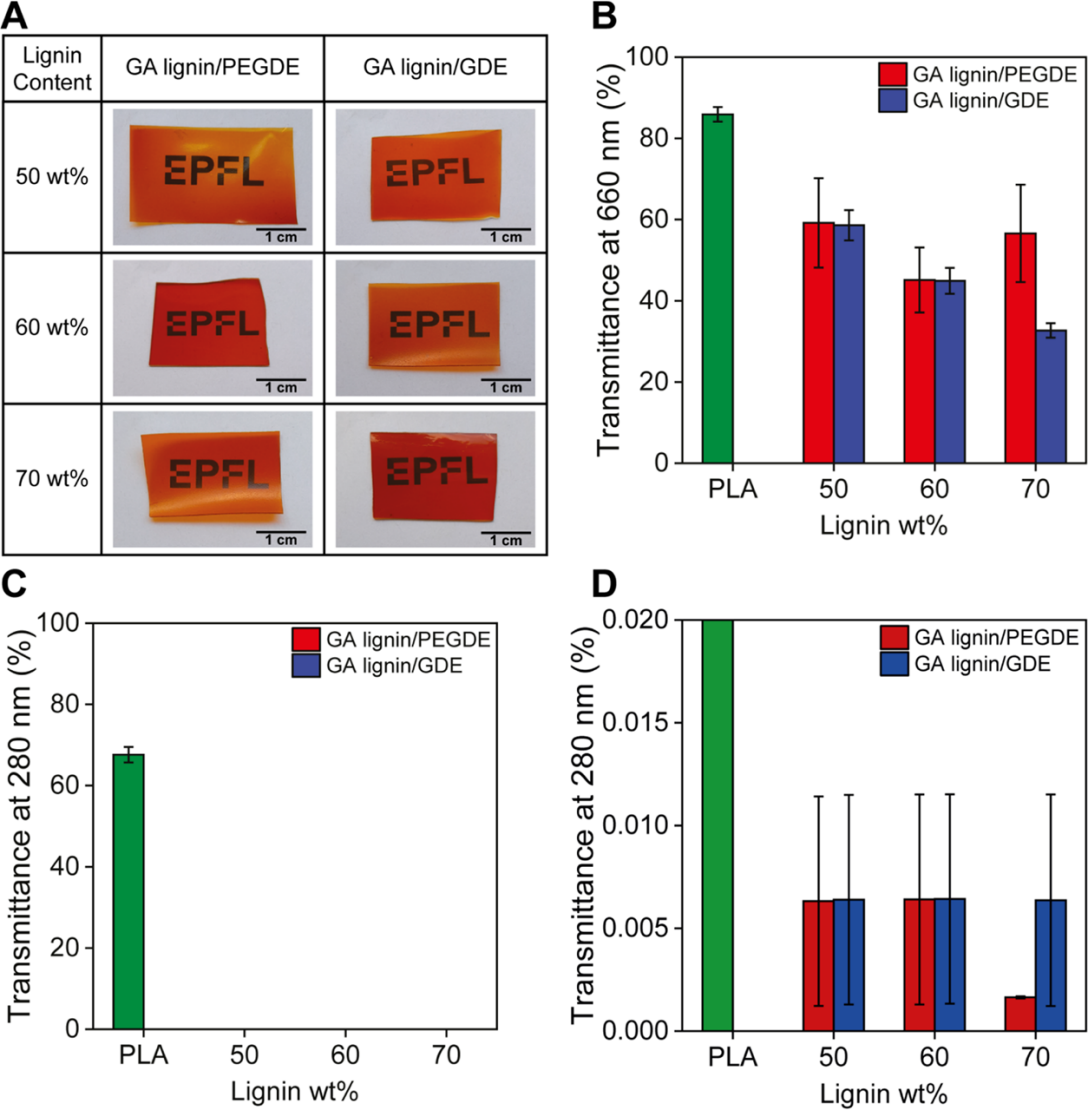
(A) Photographs of GA lignin/PEGDE and GA lignin/GDE films placed on top of a printed text to highlight their visible transparency (EPFL logo used with permission). (B) Transmittance through the films in the visible range (660 nm). (C) Transmittance through the films in the UV range (280 nm). (D) Magnification of plot (C) in the *y*-axis range 0–0.02%.

Materials used for food packaging applications need to possess adequate optical properties. On the one hand, UV light exposure promotes the degradation of vitamins, proteins, and lipids; hence, films with low transmittance in the UV range can help to prevent food from premature festering.^36^ At the same time, films with low transmittance in the visible range will be opaque and do not allow the customer to see the packaged product. The ideal food packaging material thus combines effective UV barrier properties with high transparency in the visible light region. Figure 4A shows photographs of 1 × 3 cm^2^ specimens of cross-linked GA lignin films, which were placed on top of a sheet of paper with printed text. While the films have a light brown color, the text is clearly legible, even for the films with the highest lignin content. To further highlight the transparency of the films, Supporting Information Figure S15 shows a photograph of a paperclip that was placed between GA lignin/GDE and GA lignin PEGDE films with a GA lignin content of 50 wt % and a sheet of white paper. To quantitatively characterize their optical properties, the cross-linked lignin films were analyzed by UV–vis spectroscopy. The full transmission spectra are presented in Supporting Information Figure S16, while the values of transmittance in the visible range (*T*_660_ at 660 nm) and in the UV range (*T*_280_ at 280 nm) are summarized in Figure 4B–4D. As a control, a poly(lactic acid) (PLA) film with a similar thickness was also evaluated. PLA was chosen because it is the most utilized biodegradable polymer on the food packaging market,^37^ and it is known to have high visible transparency.^38^ As illustrated in Figure 4B–4D, the lignin films have superior UV barrier properties as compared to PLA with *T*_280_ < 0.01% and a reasonable transmittance of ∼60% in the visible wavelength region. The GA lignin/GDE and GA lignin/PEGDE films have very similar UV barrier properties. All films have a *T*_280_ of less than 0.01% with no significant differences across the various samples. The visible light transparency of the GA lignin films seems to correlate with the sol fraction and thus cross-linking density (see also Figure 2). For the GA lignin/PEGDE films, no significant differences in visible light transmittance were observed, and the *T*_660_ for the GA lignin/GDE films was found to decrease by increasing the GA lignin content. The relatively high transmittance of the GA lignin films in the visible wavelength region is remarkable for lignin-based films.^39−41^ This becomes evident when films prepared from GA lignin are compared with analogues that are obtained by cross-linking soda lignin with PEGDE and GDE following the same protocol. Supporting Information Figure S17 shows the images of soda lignin films cross-linked with 50 wt % PEGDE and GDE. In contrast to films prepared with GA lignin, the soda lignin-based films are dark brown and nontransparent. These marked differences in optical properties between the different types of lignin are a direct consequence of the extraction processes that are used for their isolation. Soda lignin is extracted at high temperatures and using strong alkaline conditions, which promote the formation of chromophores, such as C=C bonds, conjugated with aromatic rings, which are normally found in industrial technical lignins and strongly absorb light in the visible range.^16^ The AAF extraction process that is used to isolate GA lignin, in contrast, uses milder conditions that prevent these reactions.

**Figure 5:**
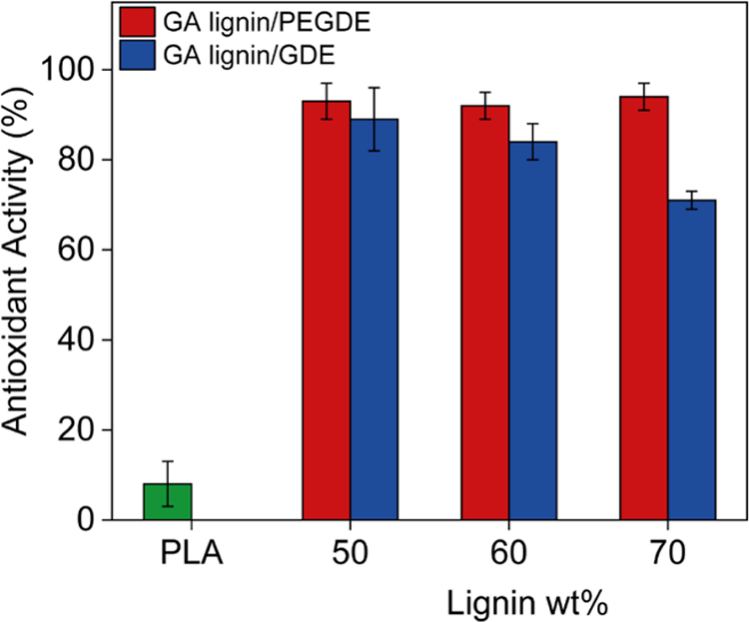
Antioxidant activity of PLA (green), as well as GA lignin/PEGDE (red) and GA lignin/GDE (blue) films containing 50, 60, and 70 wt % GA lignin, as measured by the DPPH assay.

To prevent food oxidation,^42,43^ packaging materials often incorporate antioxidants such as, for example, butylated hydroxytoluene (BHT) and butylated hydroxyanisole (BHA).^44^ The use of these compounds, however, can lead to the generation of nitrates and sulfates, which are responsible for allergies and may have other side effects on human health.^45,46^ To reduce the use of these potentially toxic compounds, natural antioxidants such as lignin can be employed.^47^ Lignin is an attractive material for food packaging applications, as it possesses antioxidant activity. The antioxidant activity of lignin is due to its high content of phenol groups, which can act as radical scavengers and inhibit oxidation reactions.^48,49^ The antioxidant activity of the GA lignin films was tested by the DPPH assay.^50^ DPPH is a stable free radical with an intense absorbance at 517 nm in a methanol solution. Once the free electron of DPPH has been paired with another radical (such as those that can be produced during oxidation processes), the DPPH solution becomes colorless. This allows us to compare the antioxidant activity of various antioxidants or materials, including lignin-containing films and fibers.^51,52^ The DPPH assay is illustrated in Supporting Information Scheme S2. In Figure 5, the antioxidant activities of the GA lignin films are presented. For these experiments, the methanolic DPPH solution was exposed to the films for 2 h and then the absorbance at 517 nm compared with that of the initial solution to determine the antioxidant activity of the samples. As for the UV–visible transmittance measurements, a PLA film was evaluated as a control sample. All of the GA lignin films showed at least four times higher antioxidant activity as compared to the PLA control. In general, the antioxidant activities of the cross-linked GA lignin films were similar, except for the samples prepared with 70 wt % lignin, where the activity of the GDE cross-linked sample was significantly lower as compared to the PEGDE cross-linked sample. For the GA lignin/PEGDE films, the antioxidant activity did not significantly vary by changing the GA lignin content. For the GA lignin/GDE films, in contrast, a small decrease in the antioxidant activity was observed by increasing the GA lignin content. This may be related to an increase in cross-linking density by increasing the GA lignin content and a concomitant reduction in the phenol group content in the GA lignin/GDE films.

**Figure 6:**
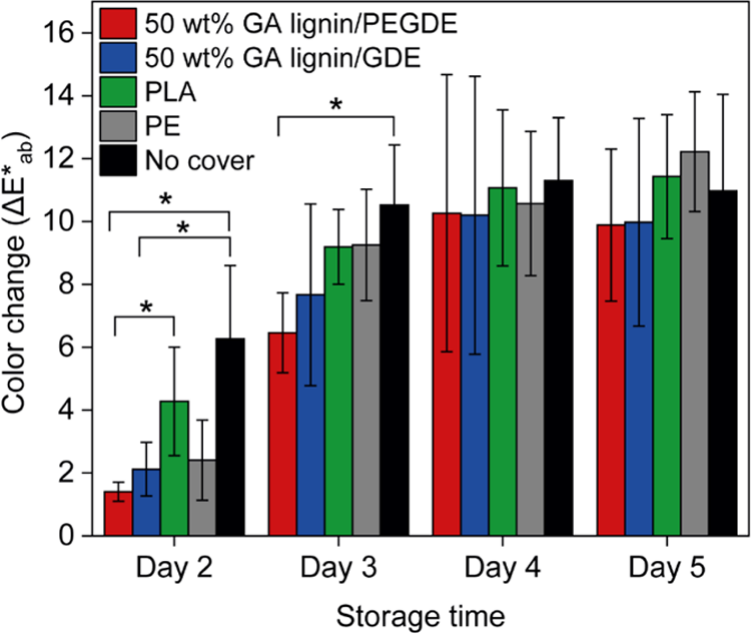
Color change (ΔE*_ab_) of broccoli florets stored under sunlight covered by a 50 wt % GA lignin/PEGDE (red), 50 wt % GA lignin/GDE (blue), PLA (green), or a polyethylene (PE) food wrapping film (gray) or not covered (black). ΔE*_ab_ represents the color change of the broccoli compared to the first day of the experiment (e.g., day 1). Statistical analysis was performed with Student’s *t*-test (*p < 0.05, ** *p* < 0.01, ***p < 0.001).

As a first proof-of-concept model experiment, the GA lignin films were evaluated as broccoli packaging films. The color of broccoli is very sensitive to UV light exposure, which can cause chlorophyll degradation^53^ and formation of carotenoids,^54,55^ resulting in yellowing of the broccoli florets. As the color change is accompanied by a loss of nutritional value, this vegetable is normally stored inside appropriate food packaging that minimizes the UV light exposure. For this experiment, broccoli florets were stored in Teflon containers, which were covered with a 50 wt % GA lignin/PEGDE, a 50 wt % GA lignin/GDE, a PLA, or a polyethylene (PE) food wrapping film. As a control, broccoli florets were also stored in a container that was not covered with a polymer film. The containers with the broccoli florets were stored under ambient conditions under exposure to sunlight, and the color of the florets was measured with a colorimeter over a period of 5 days. The colorimeter provides a quantitative measure (ΔE*_ab_) that can be used to monitor the color change of the florets as a function of time. Figure 6 presents the ΔE*_ab_ values measured for broccoli florets that were stored under the different films over a period of 5 days. The data in Figure 6 show that the GA lignin-based films perform as well as the PLA and PE films in retarding the discoloration of the broccoli florets. While the error bars are relatively large and the differences are not statistically significant, the data suggest that for a period of 3 days, the GA lignin films outperform the PLA and PE films.

### Covalently Attached GA Lignin Coatings

**Scheme 2:**
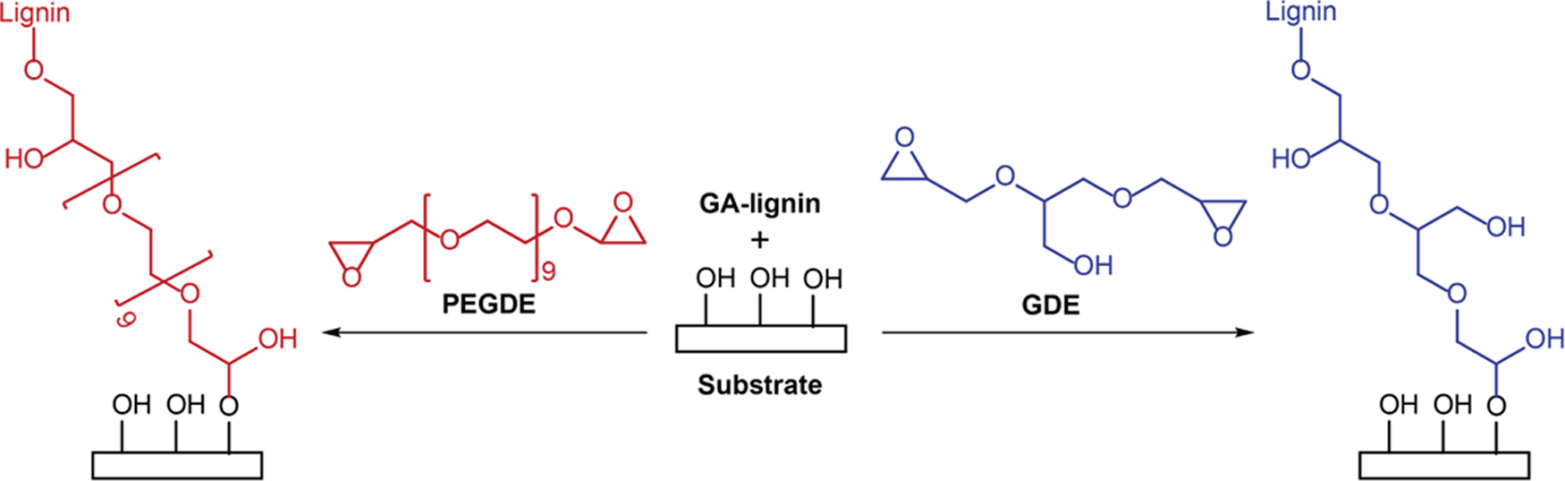
Preparation of Covalently Surface-Attached PEGDE and GDE Cross-linked GA Lignin Coatings

The GA lignin/bis-epoxide formulations discussed above not only provide access to freestanding films but potentially can also be used to covalently coat substrates, which present epoxide-reactive groups, such as carboxylic acid, alcohol, or amine groups, with a lignin-based coating (Scheme 2). This would provide an avenue to augment the UV barrier properties and antioxidant activity of the underlying substrate material. As a first proof of concept, the bis-epoxide-based cross-linker approach was explored to modify plasma-treated silicon and glass substrates, which present epoxide-reactive alcohol groups. To prepare surface-attached lignin coatings, GA lignin and PEGDE or GDE were dissolved in dioxane and the solution was deposited on a plasma-activated silicon or glass substrate. After complete evaporation of the solvent, the coatings were cured by heating at 120 °C for 3 h. Coatings were prepared from GA lignin/PEGDE or GA lignin/GDE mixtures containing 50, 70, and 90 wt % GA lignin. The resulting coatings had thicknesses of 0.13–0.16 mm and were robustly covalently attached to the substrate, as can be seen from photographic images that were taken of lignin-coated glass slides before and after immersion in dioxane for 1 h (Supporting Information Figure S18). In contrast, when films were prepared following the same procedure but with pure GA lignin, without the addition of a cross-linker, the coating was dissolved and washed from the surface (Supporting Information Figure S18). This indicates that the bis-epoxides not only act as a cross-linker but also react with surface hydroxyl groups on the silica substrate, allowing for a robust covalent attachment of the coatings.

FTIR spectra of lignin coatings that were applied on silicon wafers are presented in Supporting Information Figure S19. In the spectra, characteristic lignin peaks can be identified at 3400 cm^–1^ (O–H stretching) and 1730 cm^–1^ (C=O stretching). The FTIR spectra of the pure cross-linkers, which are also presented in the Supporting Information, Figure S19, are characterized by signals at 2860 cm^–1^ (C–H stretching), 1090 cm^–1^ (C–H bending) as well as the oxirane signal at 900 cm^–1^. The first two signals also appear in the spectra of the lignin coatings and increase in intensity when the lignin content is decreased. Similar to what was observed for the freestanding films, the oxirane peak at 900 cm^–1^ is present in the spectrum of the 50 wt % GA lignin/GDE coating, indicating that in the coatings containing the lowest lignin amount, not all of the epoxide groups have reacted.

SEM analysis of the GA lignin-coated silicon substrates revealed a smooth, uniform, and defect-free surface morphology, even for coatings prepared with 90 wt % lignin (Supporting Information Figure S20A; higher magnification SEM images are provided in Supporting Information Figure S21A). Surface coatings that were prepared following the same procedure but with soda lignin instead of GA lignin, in contrast, were characterized by a rough surface morphology and possessed cracks (Supporting Information Figures S20B and S21B).

The wettability of the coatings was assessed by water contact angle analysis (Figure S22). The water contact angles of the GA lignin/GDE surface coatings are higher than those of the corresponding GA lignin/PEGDE films, which reflects the more hydrophilic nature of the PEGDE cross-linker as compared to GDE. The water contact angle of the coatings also increases by increasing the lignin content, which is due to the hydrophobic character of this polymer (as compared to the two bis-epoxide cross-linkers).

**Figure 7:**
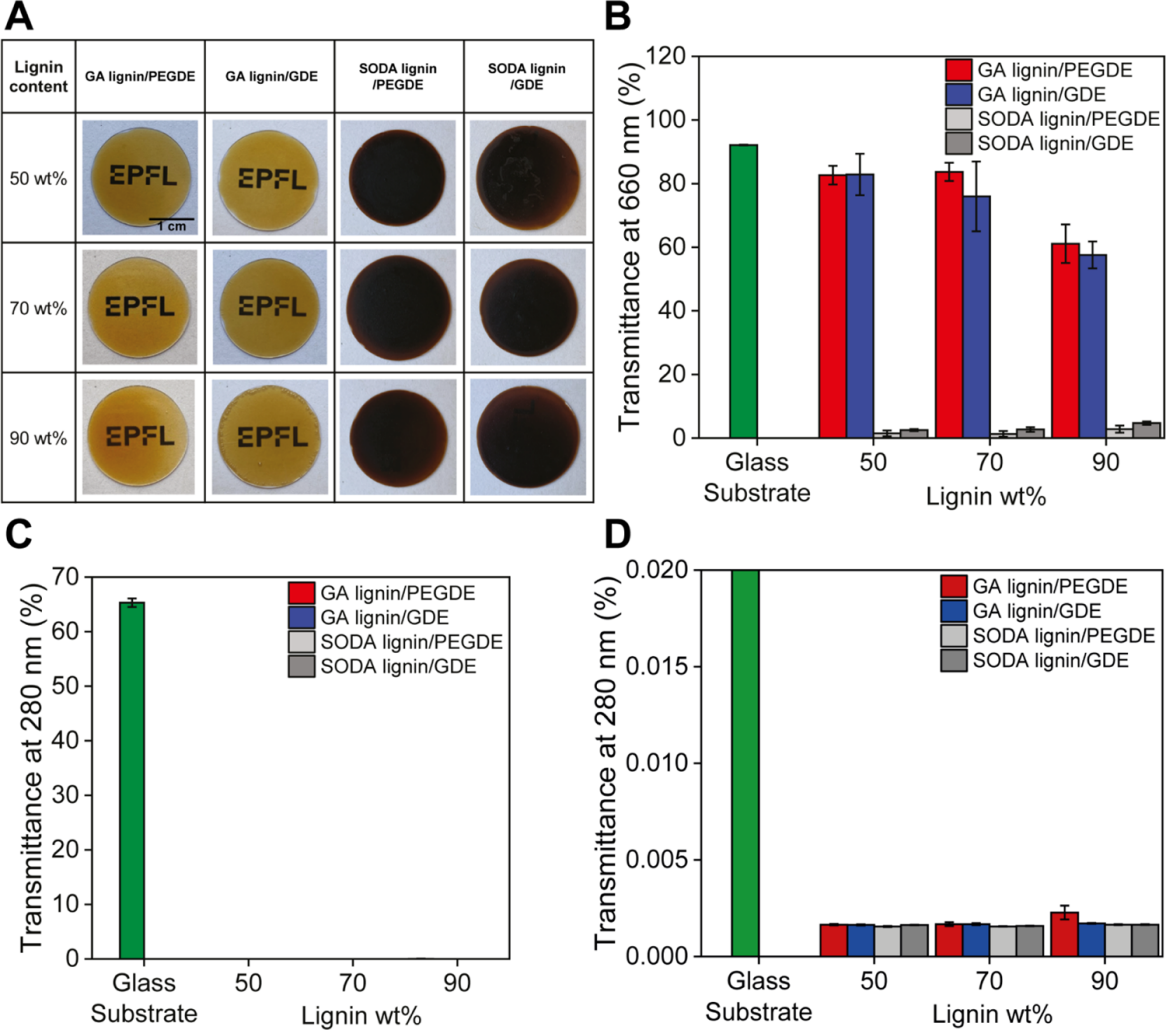
(A) Photographs of GA lignin/PEGDE, GA lignin/GDE, soda lignin/PEGDE, and soda lignin/GDE coatings on activated fused silica, overlapped on a printed text to highlight their visible transparency (EPFL logo used with permission). (B) Visible light transmittance (at 660 nm) of the different PEGDE and GDE cross-linked GA lignin and soda lignin surface coatings. (C) UV light transmittance (at 280 nm) of the different PEGDE and GDE cross-linked GA lignin and soda lignin surface coatings. (D) Magnification of plot (C) in the *y*-axis range 0–0.02%.

Similar to the freestanding films, the surface-attached GA lignin coatings combined good optical transparency in the visible light region with excellent UV barrier properties. Figure 7A presents the photographs of fused silica substrates coated with cross-linked GA lignin coatings containing 50, 70, and 90 wt % lignin, which were placed on top of a written text. All 6 coatings are optically transparent. As a control, fused silica substrates were also modified using the same protocol but with a soda-lignin-based coating. The soda-lignin-based coatings, as shown in Figure 7A, are dark and not visibly transparent. The differences between these two series of coatings highlight the unique optical properties of GA lignin. The optical properties of the GA lignin films were quantitatively characterized by measuring UV–vis transmittance spectra and determining the transmittance at 280 and 660 nm to evaluate the UV barrier properties, respectively, and visible light transparency. The UV–vis transmittance spectra of the PEGDE and GDE cross-linked GA lignin and soda-lignin-based surface coatings are included in Supporting Information Figure S23. Figure 7B–7D summarizes the transmittance of the different samples at 280 nm (*T*_280_) and 660 nm (*T*_660_). As already seen for the freestanding films, all lignin coatings exhibited strong absorption in the UV range (*T*_280_ < 0.01%). The GA lignin coatings possess a high transmittance in the visible range. The *T*_660_ gradually decreases by increasing the lignin content but remains quite high even for the 90 wt % GA lignin coatings (*T*_660_ ∼ 60%). In contrast, the soda lignin coatings exhibited very low visible light transmittance regardless of the wavelength and lignin content, with *T*_660_ < 5%. The outstanding optical properties of the GA lignin coatings underline the effectiveness of the AAF process to prevent uncontrolled condensation reactions and chromophore formation during lignin extraction.

**Figure 8:**
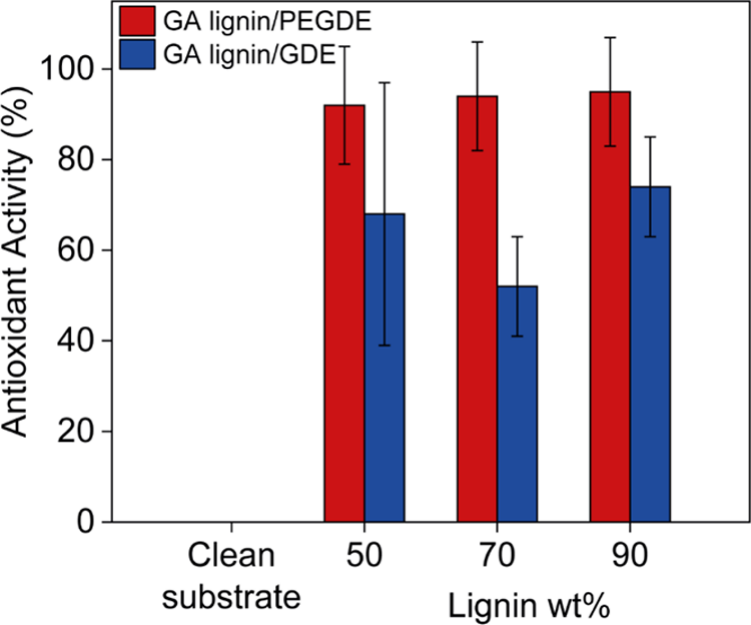
Antioxidant activity of PEGDE (red) and GDE (blue) cross-linked GA lignin-based, surface-attached coatings containing 50, 70, and 90 wt % GA lignin.

In a final series of experiments, the antioxidant activity of the GA lignin coatings was assessed by the DPPH assay. The antioxidant activity of the GA lignin/PEGDE and GA lignin/GDE coatings determined after a period of 2 h is summarized in Figure 8. As expected, the noncoated control substrate did not show any antioxidant activity, while the GA lignin-based coatings all showed antioxidant activity. No significant difference, however, can be noticed between coatings with different lignin contents.

## Conclusions

This paper has presented a simple and efficient method for the preparation of freestanding films and covalently attached surface coatings with high lignin contents (up to 70 wt % in the films and 90 wt % in the coatings). The fabrication of the films and coatings presented in this study uses cross-linkers that can be obtained from biological resources and does not require the use of catalysts or additives. The GA lignin, extracted and functionalized in one step via the AAF process in the presence of glyoxylic acid, presented high reactivity toward epoxy groups and allowed the direct preparation of thermosets via a reaction with bis-epoxy cross-linkers. The GA lignin/PEGDE and GA lignin/GDE films possess promising thermal and mechanical properties and show antioxidant activity and excellent UV barrier properties while maintaining high visible transparency, which makes them promising candidates for food packaging applications. In a first proof-of-concept experiment to explore their potential for food packaging applications, the ability of the GA lignin-based films to retard the discoloration of broccoli florets was investigated. The GA lignin films performed favorably compared to commercially available PLA and PE food packaging films that were used as controls in these experiments. The protocol for the reaction of GA lignin and bis-epoxy cross-linkers also allowed us to prepare thermoset coatings, which were covalently attached to silicon or glass substrates. The GA lignin coatings provided the substrates with excellent optical and antioxidant properties, representing an avenue to improve the performance of epoxy-reactive surfaces for application in food packaging.

## Data Availability

The source data of this study are available from the Zenodo repository at DOI: 10.5281/zenodo.10083787.

## Supporting Information Available

Description of materials and methods utilized in the study; schematic illustration of the aldehyde-assisted fractionation method to isolate lignin; weight % of GA lignin and bis-epoxide cross-linkers used to prepare freestanding films and surface-attached coatings; hydroxyl group content of lignin obtained from ^31^P NMR spectroscopy; mechanical and thermal properties of freestanding films and of commonly used polymers; HSQC and ^31^P NMR spectra; FTIR spectra; SEM images; DSC and TGA curves; transmittance spectra of freestanding films and surface-attached coatings; photographs of freestanding films prepared with soda lignin; and water contact angles of surface-attached coatings (PDF)
