# Correction to “Ductile, High-Lignin-Content Thermoset Films and Coatings”

**DOI:** 10.1021/acssuschemeng.3c07555

**Published:** 2024-01-04

**Authors:** Alice Boarino, Justine Charmillot, Monique Bernardes Figueirêdo, Thanh T. H. Le, Nicola Carrara, Harm-Anton Klok

The data availability statement of the original paper should read as follows:

## Data Availability

The source data of this study are available from the Zenodo repository at https://zenodo.org/records/10083787.

