# Supplementary material for: Ductile, High-Lignin-Content Thermoset Films and Coatings
Source: ACS Sustain Chem Eng. 2023 Nov 9;11(46):16442–52. doi: 10.1021/acssuschemeng.3c03030 (PMC10664141; doi:10.1021/acssuschemeng.3c03030)
Supplement: Supplementary file 1 — sc3c03030_si_001.pdf [file sc3c03030_si_001.pdf]

Supporting Information

Ductile, High Lignin Content Thermoset Films and Coatings

Alice Boarino,[1,±] Justine Charmillot,[2,±] Monique Bernardes Figueirêdo,[2]

*Thanh T. H. Le,[1] Nicola Carrara,[1] and Harm-Anton Klok[1]**

[1] Institut des Matériaux and Institut des Sciences et Ingénierie Chimiques, Laboratoire des Polymères, École Polytechnique Fédérale de Lausanne (EPFL), Station 12, CH-1015 Lausanne, Switzerland

[2] Bloom Biorenewables, Route de l'Ancienne Papeterie 106, CH-1723 Marly, Switzerland

alice.boarino@epfl.ch, justine@bloombiorenewables.com, monibf@gmail.com, thi.le@epfl.ch, nicola.carrara@epfl.ch, harm-anton.klok@epfl.ch

± These authors contributed equally

* Corresponding author

This Supporting Information contains:

Number of pages: 40

Number of schemes: 2

Number of tables: 6

Number of figures: 26

MATERIALS

GA lignin with a number average molecular weight (M_n) of 1300 Da, a weight average molecular weight (M_w) of 14300 Da (the gel permeation chromatography (GPC) trace is included in **Supporting Information Figure S24**), 2.83 mmol/g aliphatic alcohol groups, 1.13 mmol/g phenol groups, and 0.82 mmol/g carboxylic acid groups was provided by Bloom Biorenewables, Switzerland. GA lignin was extracted from birch wood, following a protocol described in a recent publication.[1] Soda lignin (Protobind 1000; M_n = 900 Da, M_w = 3100 Da, 2.02 mmol/g aliphatic hydroxyl groups, 1.18 mmol/g phenol groups and 0.10 mmol/g carboxylic acid groups) was supplied by Tanovis AG, Switzerland. The ^{31}P-NMR and HSQC NMR spectra of soda lignin are included in **Supporting Information Figure S25 and S26**, respectively. All reagents and solvents were used as received, unless described otherwise. 1,4-Dioxane (≥ 99.0%, Sigma-Aldrich), poly(ethylene glycol) diglycidyl ether (PEGDE) (M_n = 500 Da, Sigma-Aldrich), glycerol diglycidyl ether (GDE) (Sigma-Aldrich), deuterated chloroform (Sigma-Aldrich), chromium(III)acetylacetonate (99.99%, Sigma-Aldrich), 2-chloro-4,4,5,5-tetramethyl-1,3,2-dioxaphospholane (TMDP) (95%, Sigma-Aldrich), deuterated dimethylsulfoxide (DMSO-d6) (Sigma-Aldrich). Food packaging grade poly(lactic acid) (Ingeo biopolymer 4060D, M_w = 190'000 g/mol) was provided by NatureWorks LLC, USA.

METHODS

Gel Permeation Chromatography (GPC). GA lignin was dissolved at a concentration of 3 mg/mL in dimethylacetamide (DMAc) containing 0.1 wt% LiBr, and subsequently filtered through a 0.220 µm H-PTFE filter. GPC analysis was performed using a PSS-Polymer SECcurity[2] system (PSS Polymer Standards Service GmbH, Germany) equipped with a SECcurity[2] refractive index detector. Sample analysis was done using a GRAM precolumn of 50 mm length, and three GRAM columns of 300 mm length, all with diameter of 8 mm and particle size of 20 µm. The analyses were performed at a flow rate of 1 mL/min, and a temperature of 70 °C. 11 polymethyl methacrylate (PMMA) standards with molecular weights ranging from 602 – 1,640,000 g/mol were used for the calibration.

Phosphorus-31 Nuclear Magnetic Resonance (^{31}P-NMR) Spectroscopy. The three following solutions were prepared prior to the analysis: 1.6:1 (v/v) pyridine:chloroform-d (solution A), 19 mg/mL cyclohexanol in 1.6:1 (v/v) pyridine:chloroform-d (solution B), and 11.4 mg/mL chromium(III)acetylacetonate in 1.6:1 (v/v) pyridine:chloroform-d (solution C). For the analysis, 30 mg sample was dissolved in a mixture consisting of 0.5 mL solution A, 0.2 mL solution B, and 50 µL solution C. Once dissolved, 50 µL 2–chloro–4,4,5,5–tetramethyl–1,3,2–dioxaphospholane (TMPD) was added as the phosphorylating agent. The NMR spectra were recorded on a Bruker Avance NEO 400 MHz spectrometer with a 10 second relaxation delay and 256 scans. The signal of the TMDP reacted with water (humidity) was used as the reference (132.3 ppm).

^{1}H-^{13}C Heteronuclear single quantum coherence (HSQC) spectroscopy. 60 mg sample was dissolved in 0.5 mL of deuterated DMSO. HSQC spectra were recorded on a Bruker Avance NEO 400 MHz spectrometer acquiring 4 scans and using 1.5 seconds of relaxation delay. The residual solvent signal was used as the reference (δH/δC 2.50/39.5 ppm).

Fourier Transform Infrared spectroscopy (FTIR). FTIR spectra were acquired on a Nicolet 6700 instrument from ThermoFischer Scientific, from 650 to 4000 cm^{-1}, using on average 32 scans.

Scanning electron microscopy (SEM). SEM images of the films and coatings were acquired on a Zeiss GeminiSEM 300 at 3.00 kV. Before the analyses, the samples were coated with a 10 nm protective layer of Au/Pd on a Q150T Plus Turbomolecular Pumped Coater (Quorum Technologies).

Mechanical testing. The mechanical properties of the freestanding films were measured using an AUTOGRAPH Table-TOP Precision Universal Tester from Shimadzu, equipped with a 10 kN load cell. Experiments were performed using ASTM D638 Type IV dog bone-shaped samples, prepared in a mold with the required shape, with a length and a width of narrow section of 33 and 6 mm, respectively. At least three specimens were tested for each sample at a constant speed of 5 mm/min. All the dog bones had an average thickness of 0.3 - 0.5 mm, which was measured with a micrometer in at least three

different regions of the specimen. The sample toughness was determined as the area under the stress–strain curve.

Thermal analysis. Thermogravimetric analysis (TGA) was conducted using a Perkin Elmer TGA 400 instrument. TGA analysis was performed in air by increasing the temperature from 30 to 900 °C with a heating rate of 10 °C/min. Differential Scanning Calorimetry (DSC) measurements were performed on a TA Instruments DSC Q100. The samples were first heated from -70 to 200 °C at a rate of 10 °C/min to erase any previous thermal history, then cooled to -70 °C at a rate of 5 °C/min, and heated again from -70 to 200 °C at the rate of 10 °C/min. The second heating scan was considered to obtain the DSC curves and to determine glass transition temperatures (T_g).

Ultraviolet–visible (UV/vis) spectroscopy. A PerkinElmer Lambda 365 UV-Vis spectrophotometer was used to record the transmission spectra of the samples over the visible and the ultra-violet range, from 200 nm to 700 nm.

Coating stability test. Each coated substrate was immersed vertically in dioxane until half of the surface was covered with solvent. After 1 h, the coatings were taken out and dried under a nitrogen flow. Photographs of the coatings were taken before and after immersion. As control, a coating was prepared using only GA lignin, without any crosslinker.

DPPH colorimetric assay. The antioxidant activity of the samples was evaluated using the DPPH colorimetric assay.[2] 20 mg of freestanding film, or a 1 x 1 cm^2 coated substrate, were immersed in 2 mL of a 25 mg/L 2,2-diphenyl-1-picrylhydrazyl (DPPH) solution in methanol. After 2 hours of shaking in the dark, the absorbance spectrum of the DPPH solution was recorded. DPPH solutions in which no sample was immersed were analyzed as control. The DPPH radical-scavenging activity, or antioxidant activity, is calculated as:

$$\text{Antioxidant Activity (\%)} = \frac{(A_0\text{-}A_t)}{A_0} \times 100$$

where A_0 and A_t are the values of absorbance at 517 nm of the DPPH solution at time zero and of the DPPH solution exposed to the films or coatings for 2 hours.

Water contact angle measurements. An EasyDrop instrument from Krüss was used to measure water contact angles. 3 μL droplets of distilled water were deposited on the coated surface. The contact angle was measured within 60 seconds after deposition of the droplet. The contact angle was evaluated at three different positions for each surface and the average value reported.

Broccoli floret storage and color determination. Broccoli florets were stored at room temperature under sunlight inside a Teflon container covered with a 50 wt% GA lignin/PEGDE film, a 50 wt% GA lignin/GDE film, a PLA film, or a polyethylene (PE) food wrapping film. Non-covered samples were tested as reference. The color of the broccoli florets was analyzed using a colorimeter (CS-10 Colorimeter, CHNSpec, China). CIELAB color space was used to express the observed color. CIELAB works by representing colors in a three-dimensional space using three color-opponent channels: lightness (L*), a green-red axis (a*), and a blue-yellow axis (b*). The L* channel represents the lightness or darkness of a color, ranging from 0 (black) to 100 (white). The a* channel represents the green-red axis, with positive values indicating a greenish color and negative values indicating a reddish color. The b* channel represents the blue-yellow axis, with positive values indicating a yellowish color and negative values indicating a bluish color. Differences in color can be evaluated by analyzing ΔE^*_{ab}, which represents the distance between two colors in the three-dimensional L*a*b* space and provides a single value that summarizes the difference between the two colors. ΔE is given by:

$$\Delta E^*_{ab} = \sqrt{\left(L_t^* - L_1^*\right)^2 + \left(a_t^* - a_1^*\right)^2 + \left(b_t^* - b_1^*\right)^2}$$

where L_1^*, a_1^* and b_1^* are recorded the first day of the experiment, and L_t^*, a_t^* and b_t^* are recorded after a certain time t. A value of $\Delta E^*_{ab} \approx 2.3$ corresponds to a just noticeable difference.[3] The color of the broccoli florets was analyzed daily. The data were acquired on at least three broccoli florets, which were stored in each condition for 5 days. The color was measured on at least three points per each floret.

Table S1. Weight % (wt%) of GA lignin and bis-epoxide crosslinkers, and the corresponding molar ratios of reactive groups used to prepare the freestanding films.

Sample	GA lignin wt%	PEGDE wt%	GDE wt%	mol epoxide groups / (mol lignin COOH + aliphatic and phenol OH groups)
50 wt% GA lignin /PEGDE	50	50	-	1.1
60 wt% GA lignin /PEGDE	60	40	-	0.7
70 wt% GA lignin /PEGDE	70	30	-	0.4
50 wt% GA lignin /GDE	50	-	50	2.5
60 wt% GA lignin /GDE	60	-	40	1.7
70 wt% GA lignin /GDE	70	-	30	1.1

Table S2. Weight % (wt%) of GA lignin and bis-epoxide crosslinkers, and the corresponding molar ratios of reactive groups used to prepare the surface-attached coatings.

Sample	GA lignin wt%	PEGDE wt%	GDE wt%	mol epoxide groups / (mol lignin COOH + aliphatic and phenol OH groups)
50 wt% GA lignin /PEGDE	50	50	-	1.1
70 wt% GA lignin /PEGDE	70	30	-	0.4
90 wt% GA lignin /PEGDE	90	10	-	0.14
50 wt% GA lignin /GDE	50	-	50	2.5
70 wt% GA lignin /GDE	70	-	30	1.1
90 wt% GA lignin /GDE	90	-	10	0.36

Table S3. Aliphatic alcohol, syringyl phenol, guaiacyl phenol and carboxylic acid group content of GA lignin, and of the products obtained after reaction of GA lignin with equal amounts of PEGDE or GDE.

Sample	Aliphatic OH [mmol/g]	Syringyl OH [mmol/g]	Guaiacyl OH [mmol/g]	Carboxylic acid [mmol/g]
GA lignin	2.83	0.76	0.37	0.82
50 wt% GA lignin/PEGDE	6.89	0.73	0.37	0.49
50 wt% GA lignin/GDE	14.14	0.61	0.35	0.25

Table S4. Thickness, tensile strength, Young's modulus and toughness of GA lignin/PEGDE and GA lignin/GDE films containing 50, 60 and 70 wt% GA lignin.

Sample	Thickness (mm)	Tensile strength (MPa)	Elongation at break (%)	Young's modulus (GPa)	Toughness (MJ/m^3)
50 wt% GA lignin /PEGDE	0.41 ± 0.13	1.5 ± 0.5	114 ± 26	0.01 ± 0.005	0.32 ± 0.04
60 wt% GA lignin /PEGDE	0.27 ± 0.15	4.5 ± 0.8	235 ± 38	0.06 ± 0.02	2.88 ± 0.11
70 wt% GA lignin /PEGDE	0.32 ± 0.26	11.8 ± 0.4	149 ± 17	0.7 ± 0.4	13.31 ± 0.92
50 wt% GA lignin /GDE	0.39 ± 0.02	3.5 ± 0.5	162 ± 18	0.03 ± 0.007	2.45 ± 0.15
60 wt% GA lignin /GDE	0.39 ± 0.09	4.4 ± 1	122 ± 19	0.05 ± 0.003	2.68 ± 0.31
70 wt% GA lignin /GDE	0.40 ± 0.01	16.9 ± 3	11.4 ± 0.2	2.6 ± 0.5	2.59 ± 0.22

Table S5. Glass transition temperatures (T_g), obtained by DSC, and initial thermal degradation temperatures ($T_{5\%}$), obtained by TGA, of GA lignin, as well as GA lignin/PEGDE and GA lignin/GDE films containing 50, 60 and 70 wt% GA lignin.

Sample	T_g (°C)	$T_{5\%}$ (°C)
GA lignin	77	210
50 wt% GA lignin/PEGDE	-23	236
60 wt% GA lignin/PEGDE	1	205
70 wt% GA lignin/PEGDE	64	193
50 wt% GA lignin/GDE	-5	249
60 wt% GA lignin/GDE	17	202
70 wt% GA lignin/GDE	63	181

Table S6. Tensile strength, elongation at break, T_g and $T_{5\%}$ for polymers commonly applied in food packaging, reported as control materials in **Supporting Information Figure S13** and **Supporting Information Figure S14**.

Polymer	Tensile strength (MPa)	Elongation at break (%)	T_g (°C)	$T_{5\%}$ (°C)	Reference
PP[a]	34	200	-20	384	[4,5]
NBR[b]	15	350	-30	360	[6,7]
PLA[c]	37	4	55	309	[8]
PET[d]	55	250	80	395	[4,9]
LDPE[e]	10	600	-120	459	[10,11]
PS[f]	46	3	100	334	[4,12]
PBR[g]	12.5	300	-75	375	[6,13]
PUR[h]	25	375	-42.5	273	[6,14]

[a] Poly(propylene) (PP), [b] nitrile butadiene rubber (NBR), [c] poly(lactic acid) (PLA), [d] poly(ethylene terephthalate) (PET), [e] low density polyethylene (LDPE), [f] polystyrene (PS), [g] polybutadiene rubber (PBR), [h] polyurethane rubber (PUR).

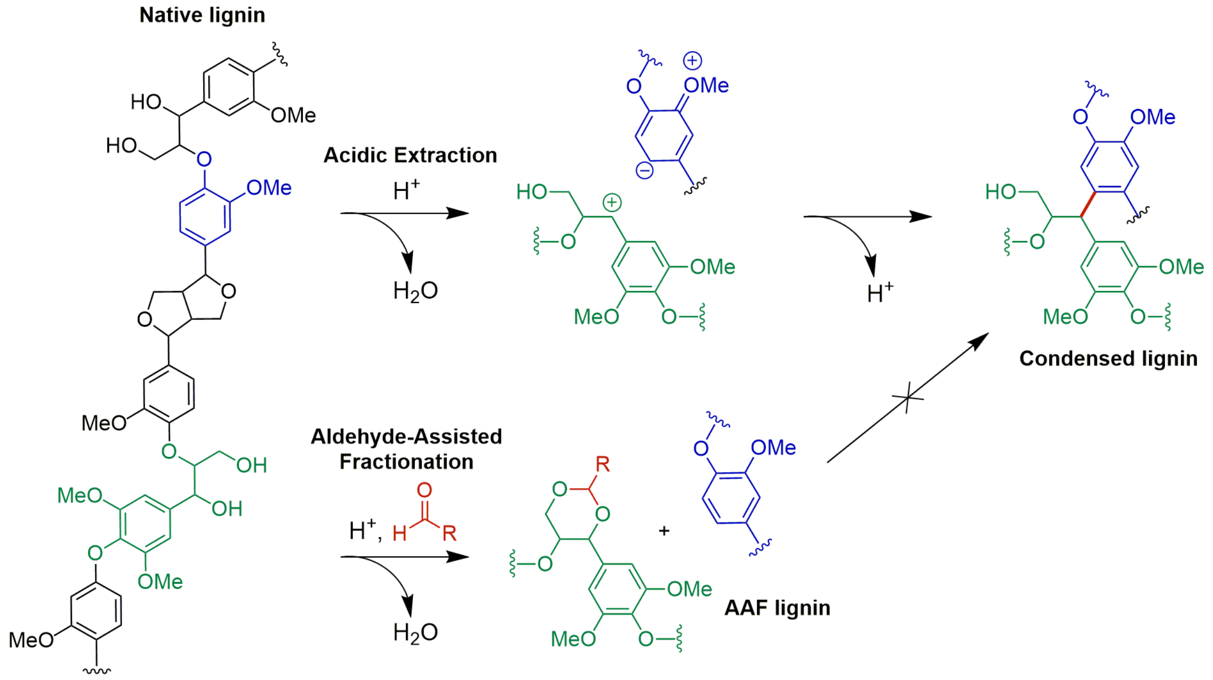

Scheme S1. Illustration of the traditional acidic lignin extraction method (top) and the aldehyde-assisted fractionation (AAF) pathway to isolate lignin.

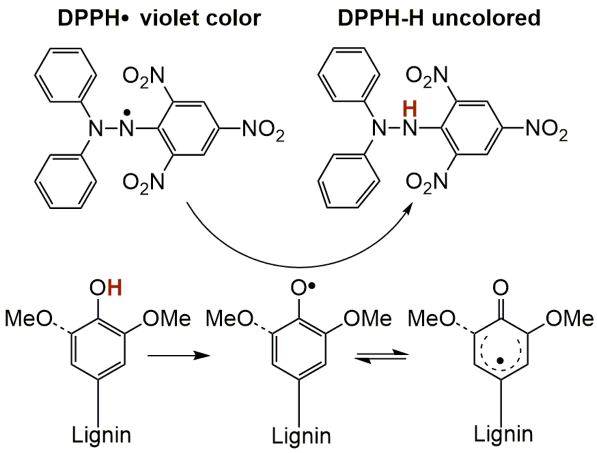

Scheme S2. DPPH reaction scheme. Dashed lines indicate the facultative presence of the methoxyl groups, resulting in either syringyl or guaiacyl units.

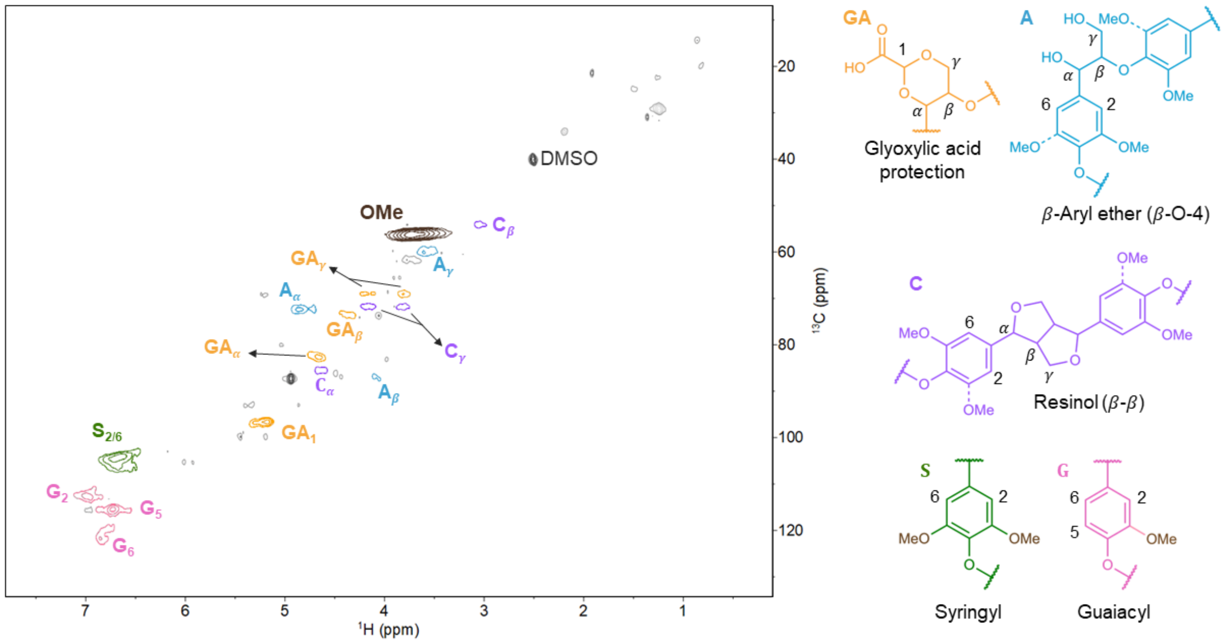

Figure S1. HSQC spectrum of GA lignin recorded by dissolving 60 mg of sample in 0.5 mL deuterated DMSO, with the corresponding lignin structures that were assigned to the signals. The unlabeled grey areas correspond to impurities or unresolved signals.

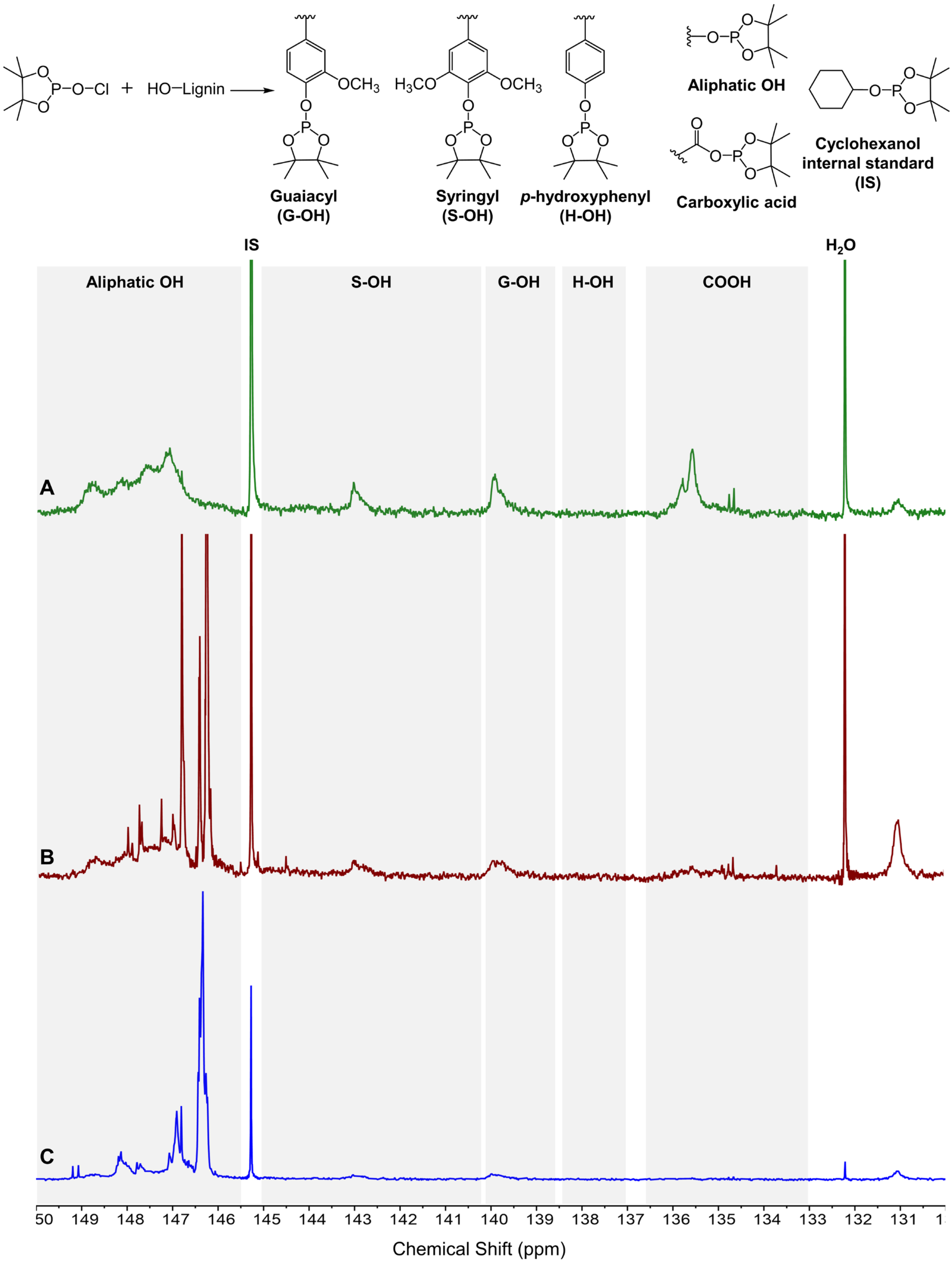

Figure S2. ^{31}P-NMR spectra of **A)** GA lignin, **B)** GA lignin after reaction with an equal amount of PEGDE and **C)** GA lignin after reaction with an equal amount of GDE.

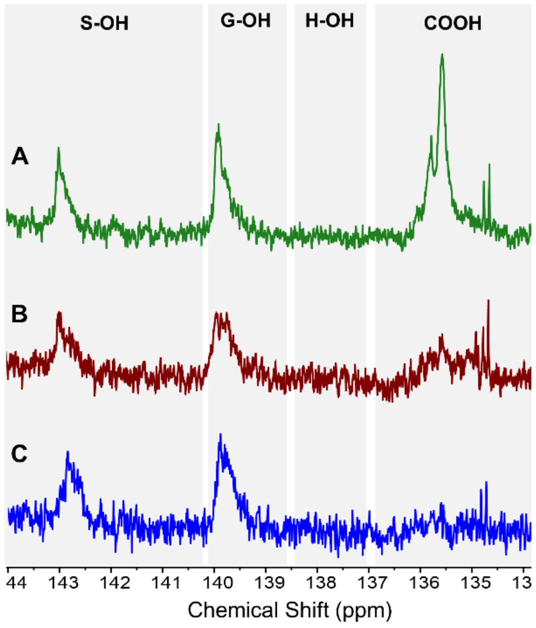

Figure S3. Magnification in the chemical shift range 134 – 144 ppm of the ^{31}P-NMR spectra of **A)** GA lignin, **B)** GA lignin after reaction with an equal amount of PEGDE and **C)** GA lignin after reaction with an equal amount of GDE.

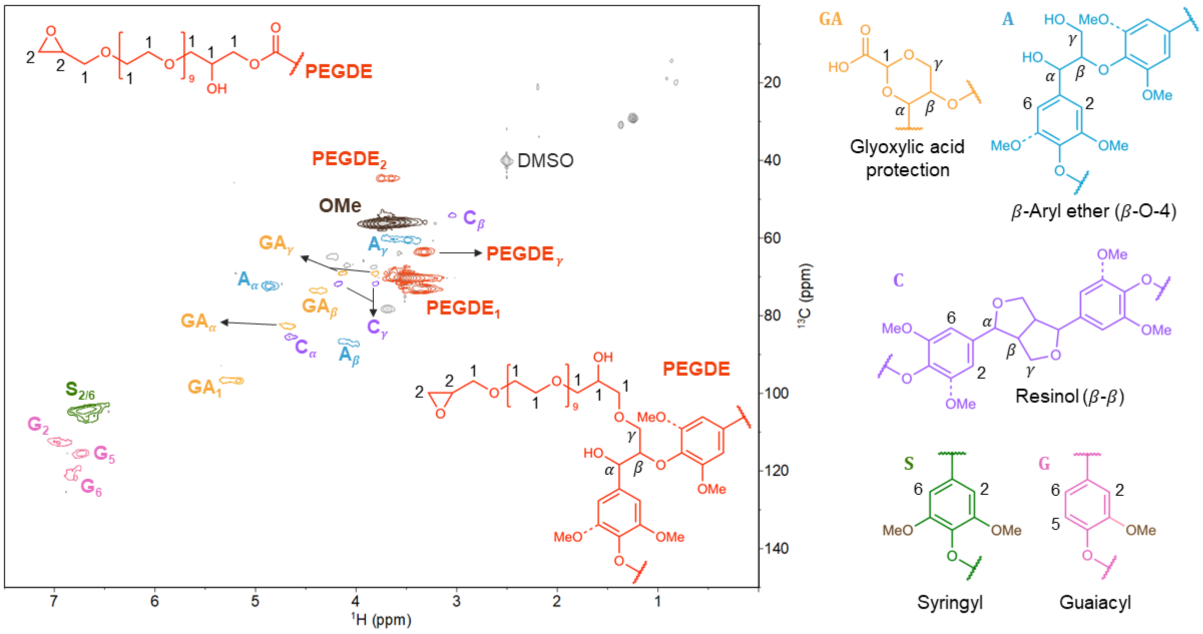

Figure S4. HSQC spectrum of the GA lignin modified with PEGDE, recorded by dissolving 60 mg of sample in 0.5 mL deuterated DMSO, with the corresponding lignin structures that were assigned to the signals. The unlabeled grey areas correspond to impurities or unresolved signals.

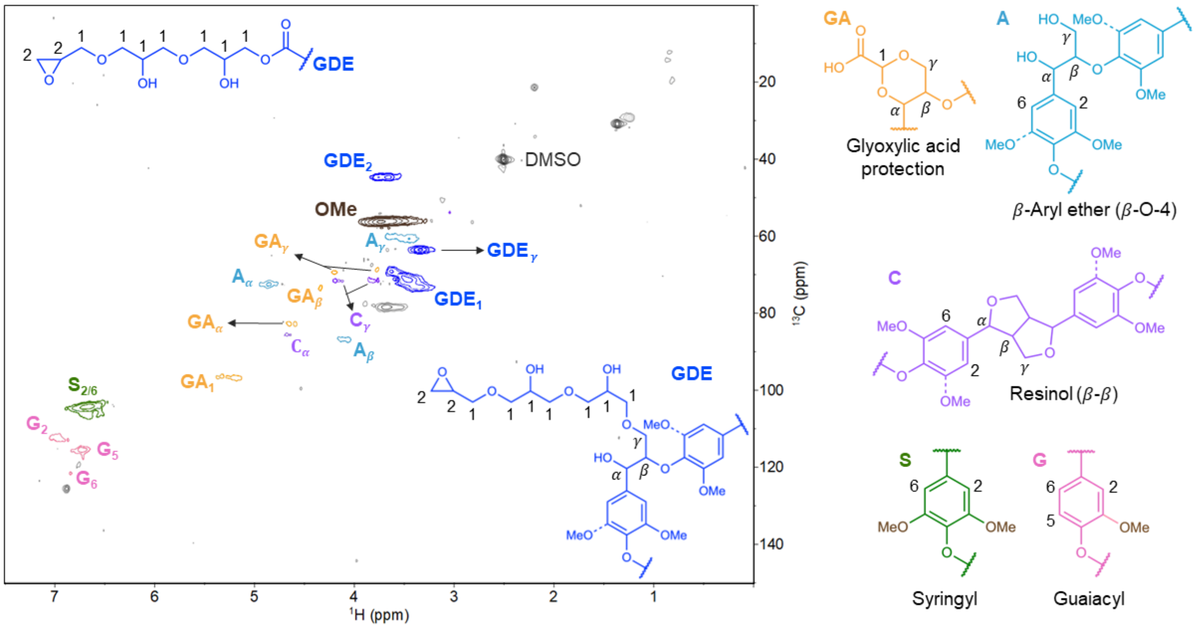

Figure S5. HSQC spectrum of the GA lignin modified with GDE, recorded by dissolving 60 mg of sample in 0.5 mL deuterated DMSO, with the corresponding lignin structures that were assigned to the signals. The unlabeled grey areas correspond to impurities or unresolved signals.

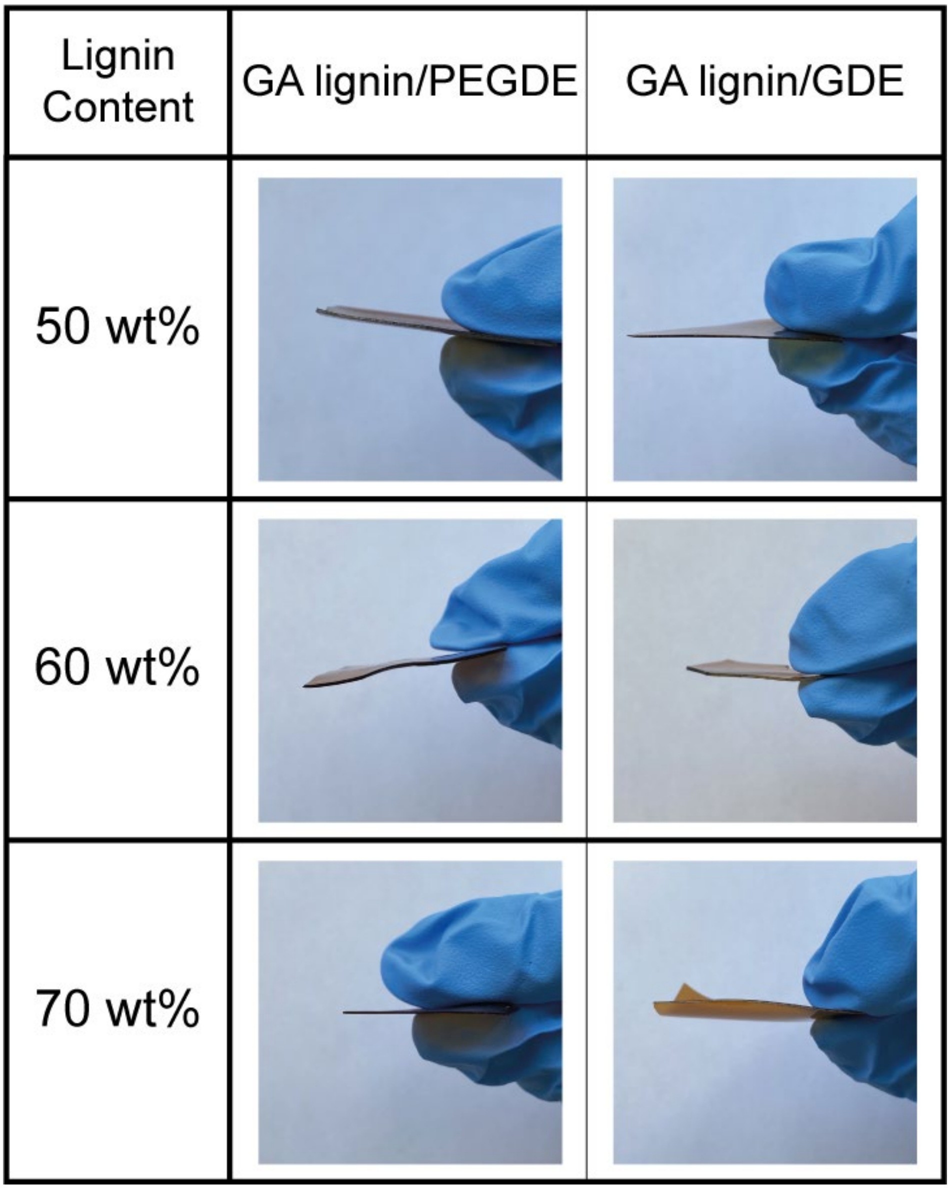

Figure S6. Photographs of GA lignin/PEGDE and GA lignin/GDE films containing 50, 60 or 70 wt% lignin.

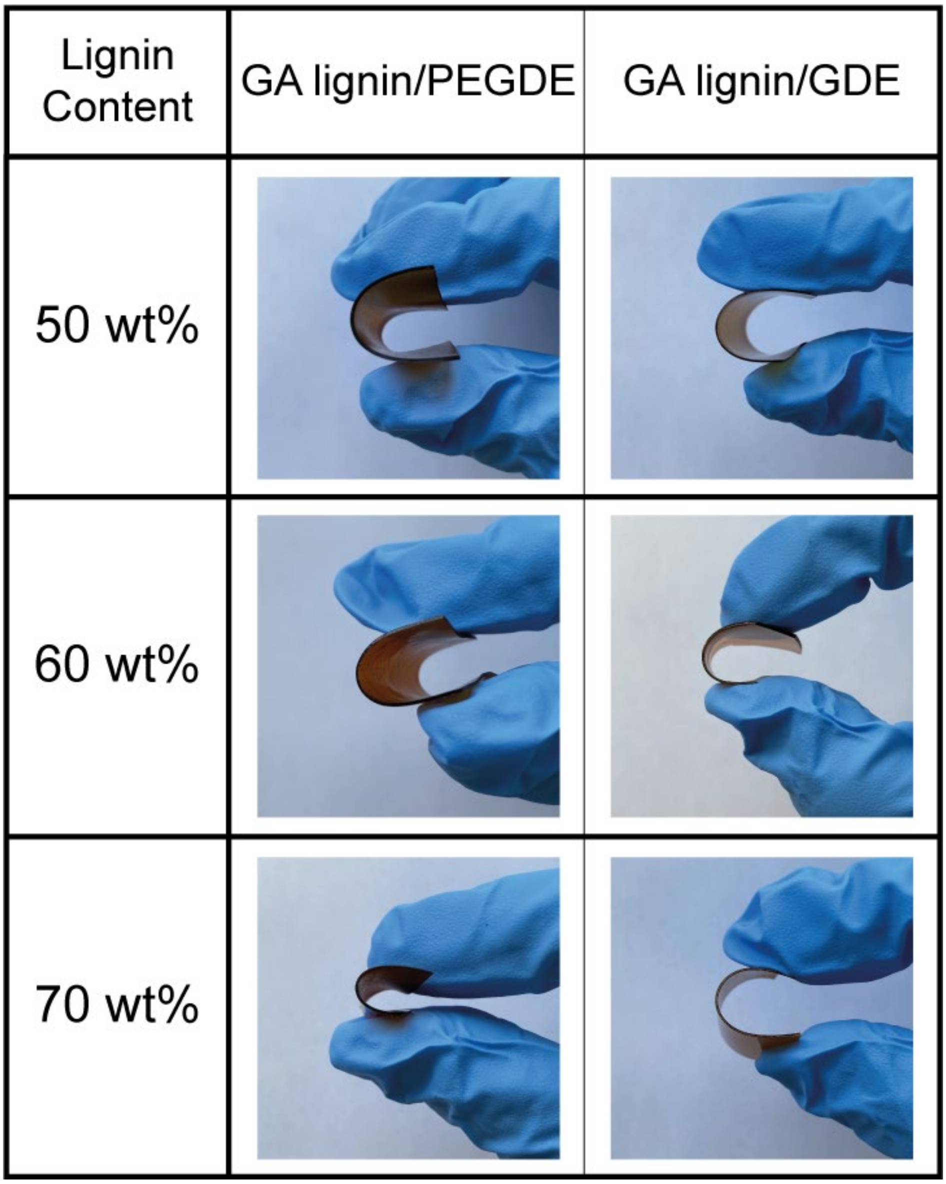

Figure S7. Photographs of GA lignin/PEGDE and GA lignin/GDE films containing 50, 60 or 70 wt% lignin, which were bent to highlight their flexibility.

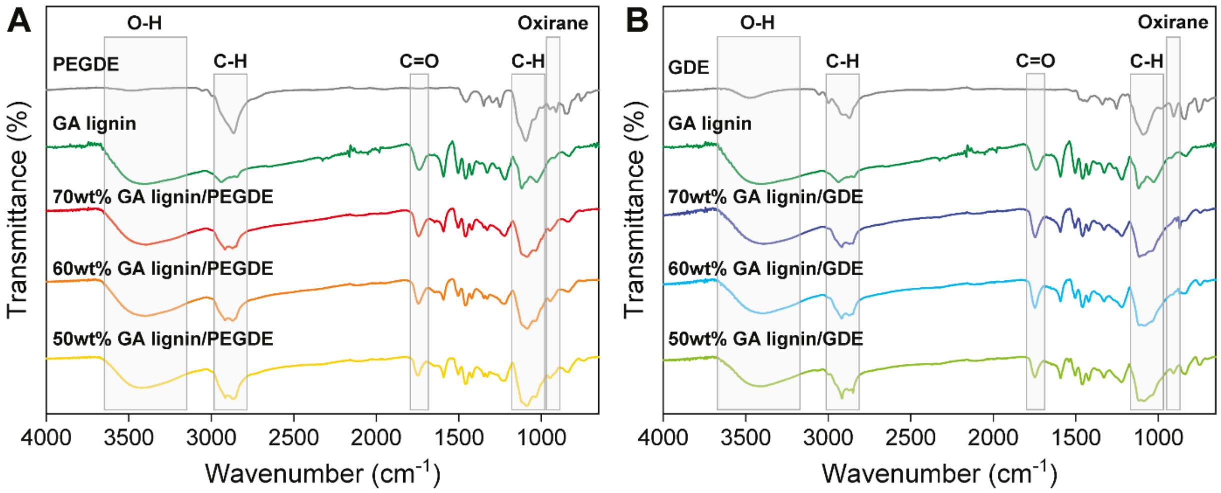

Figure S8. FTIR spectra of **A)** GA lignin/PEGDE and **B)** GA lignin/GDE films containing 50, 60 and 70 wt% GA lignin. The spectra of pure PEGDE, GDE and GA lignin are included for comparison.

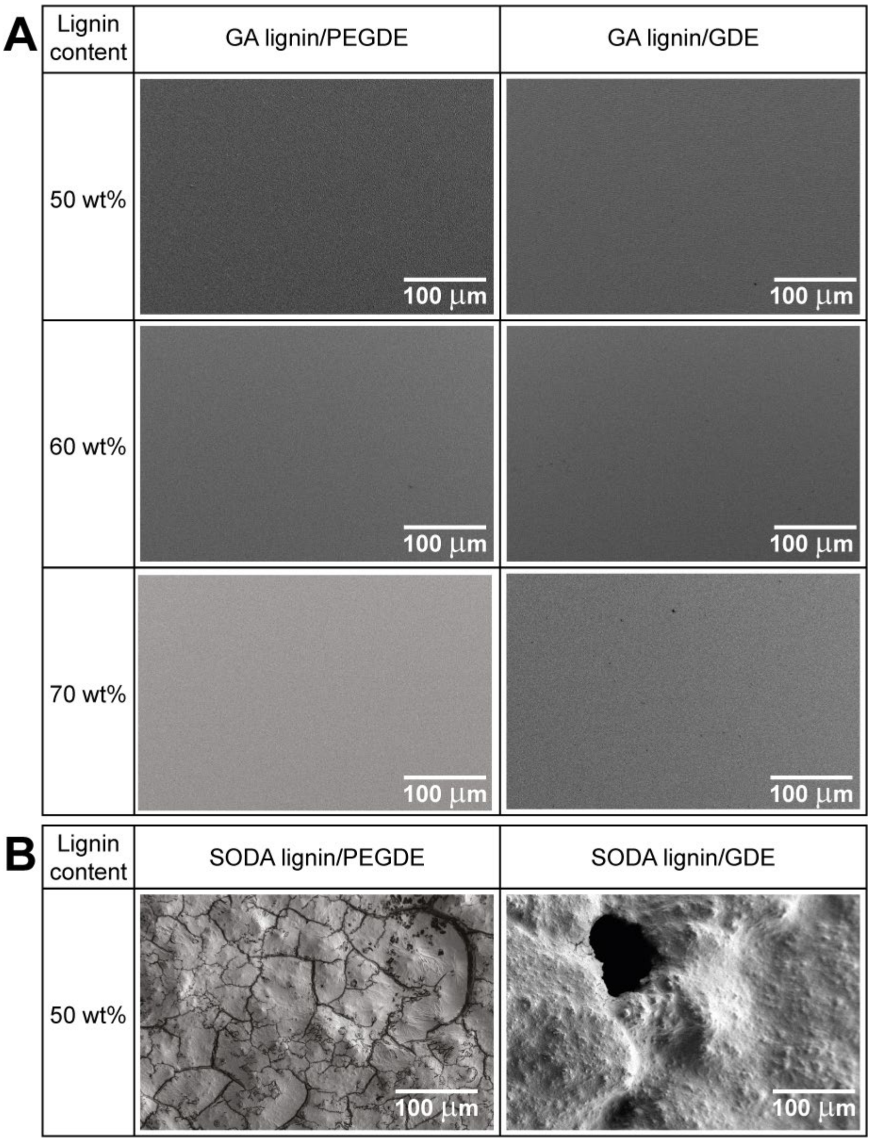

Figure S9. SEM images of **A)** GA lignin films prepared by crosslinking with PEGDE and GDE, and **B)** Soda lignin films prepared by crosslinking with PEGDE and GDE.

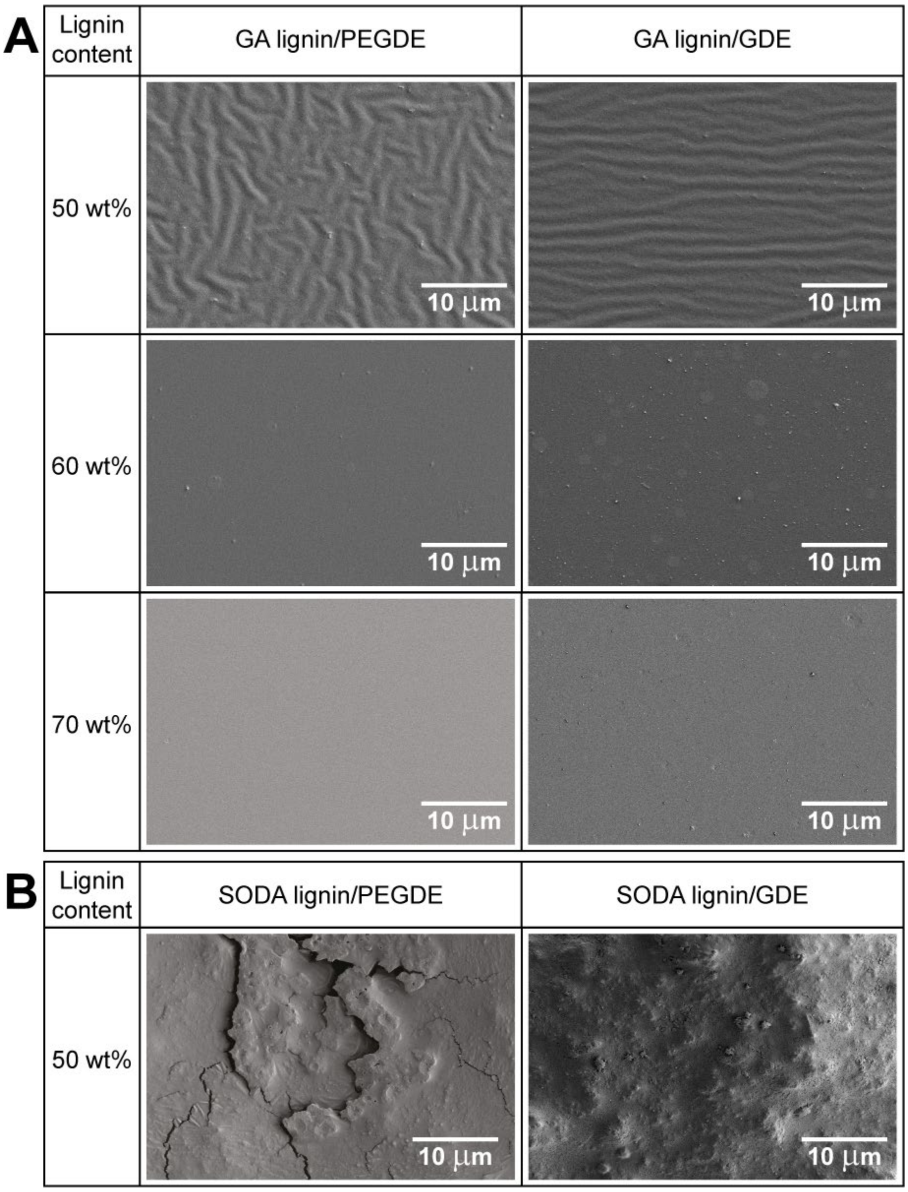

Figure S10. SEM images of **A)** GA lignin films prepared by crosslinking with PEGDE and GDE, and **B)** Soda lignin films prepared by crosslinking with PEGDE and GDE.

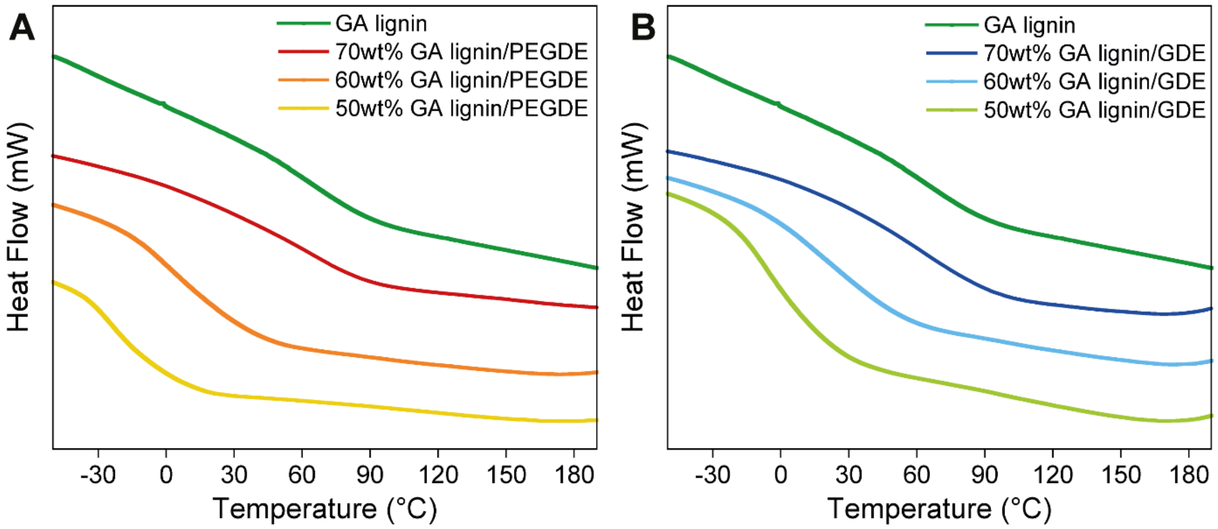

Figure S11. DSC curves (endo up) of **A)** GA lignin/PEGDE and **B)** GA lignin/GDE films containing 50, 60 and 70 wt% GA lignin.

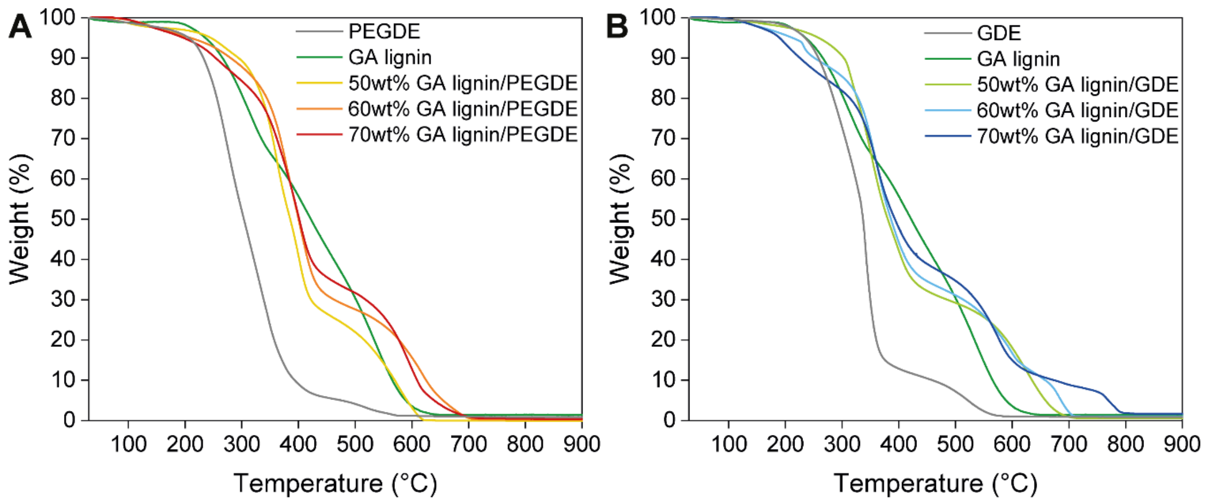

Figure S12. TGA curves of **A)** GA lignin/PEGDE and **B)** GA lignin/GDE films containing 50, 60 and 70 wt% GA lignin. TGA curves of pure GA lignin, PEGDE and GDE are included as well.

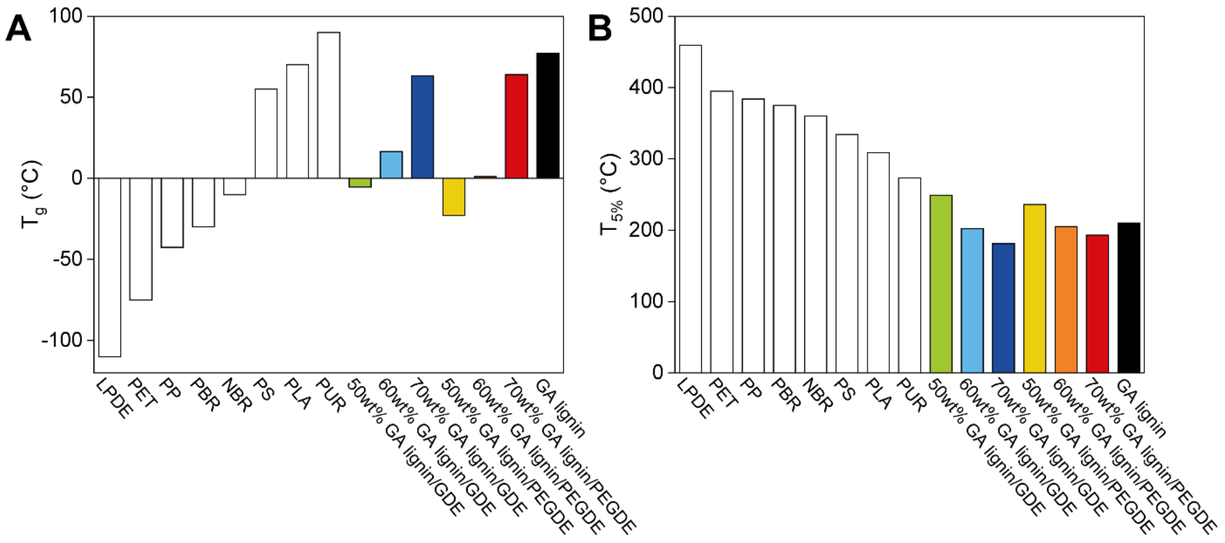

Figure S13. Thermal properties of the crosslinked GA lignin films, compared to other commercial plastics: **A)** glass transition temperature (T_g) and **B)** initial thermal degradation temperature ($T_{5\%}$) (temperature at which the mass of the sample is 5% less than its mass at 50°C). Detailed values of T_g and $T_{5\%}$ of the control polymers, as well as the references from which they were taken, are reported in **Supporting Information Table S6**.

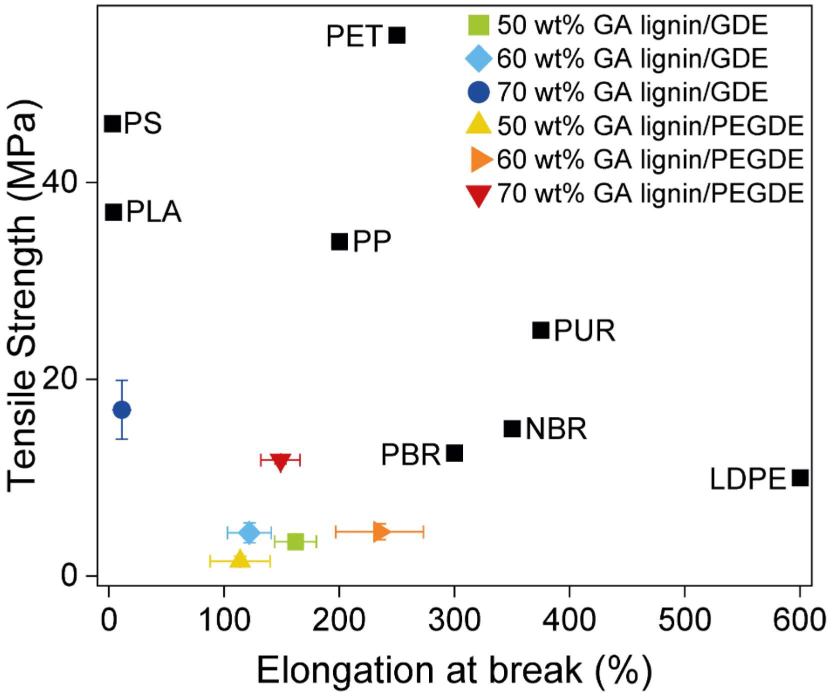

Figure S14. Average tensile strength versus average elongation at break of the crosslinked GA lignin films, compared to other commercial plastics: poly(ethylene terephthalate) (PET), polystyrene (PS), poly(lactic acid) (PLA), poly(propylene) (PP), polyurethane rubber (PUR), nitrile butadiene rubber (NBR), polybutadiene rubber (PBR) and low density polyethylene (LDPE). Detailed values of tensile strength and elongation at break of the control polymers are reported in **Table S6**.

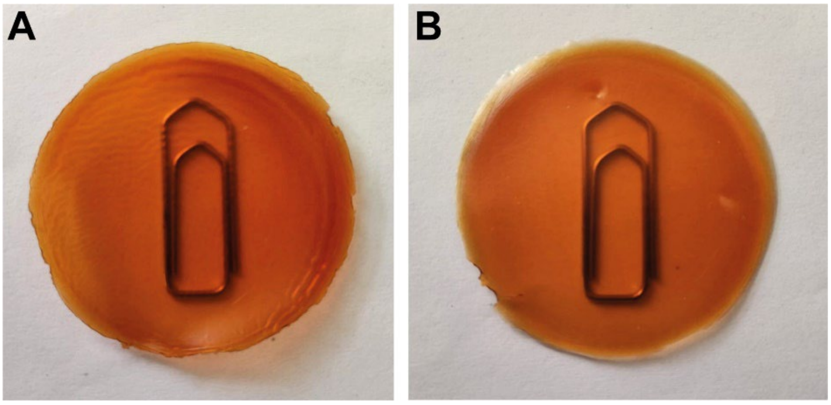

Figure S15. Pictures of a paperclip, which was placed between a white paper and **A)** GA lignin/PEGDE and **B)** GA lignin/GDE films containing 50 wt% lignin.

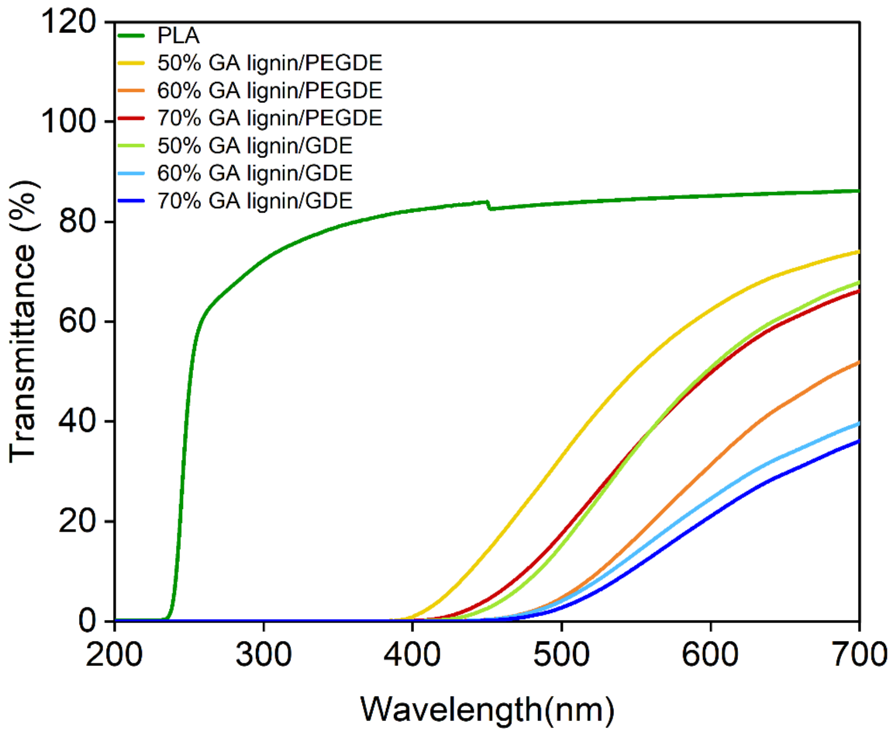

Figure S16. Transmittance spectra recorded from 200 nm to 700 nm of GA lignin/PEGDE and GA lignin/GDE films containing 50, 60 and 70 wt% GA lignin. The spectrum of a PLA film of similar thickness is included for comparison.

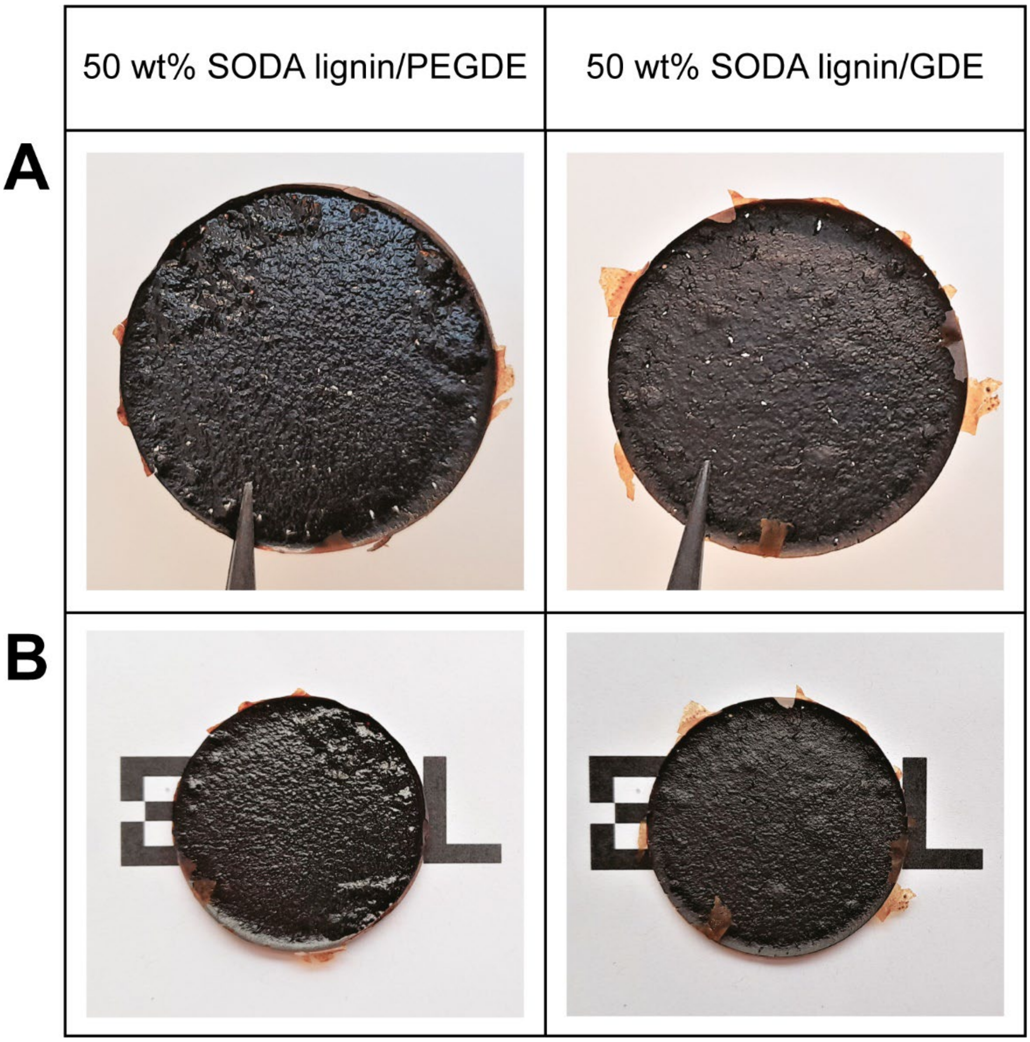

Figure S17. A) Photographs of freestanding films prepared with 50 wt% Soda lignin and PEGDE or GDE. **B)** Films prepared with 50 wt% Soda lignin and PEGDE or GDE overlapped on a printed text to highlight their opacity (EPFL logo used with permission).

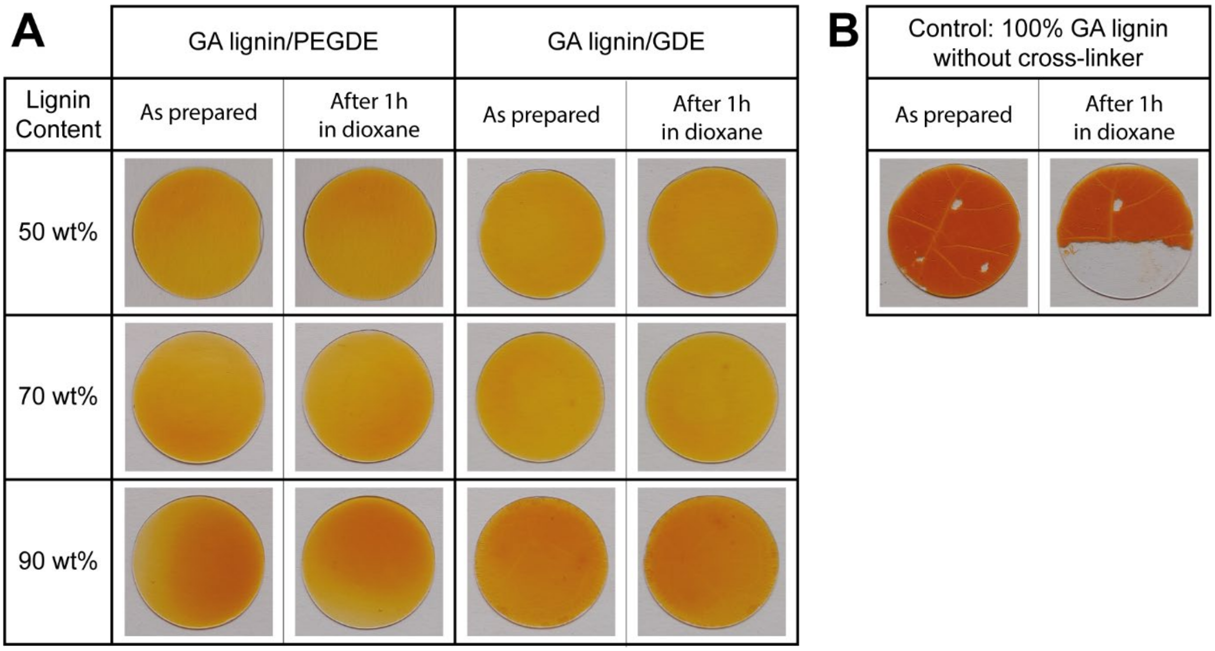

Figure S18. Photographs of **A)** GA lignin/PEGDE and GA lignin/GDE coatings containing 50, 70 or 90 wt% lignin before and after immersion in dioxane for 1 hour and dried under a flow of nitrogen; **B)** Photographs of a coating prepared from 100% GA lignin, without addition of any cross-linker, before and after immersion in dioxane for 1 hour and dried under a flow of nitrogen.

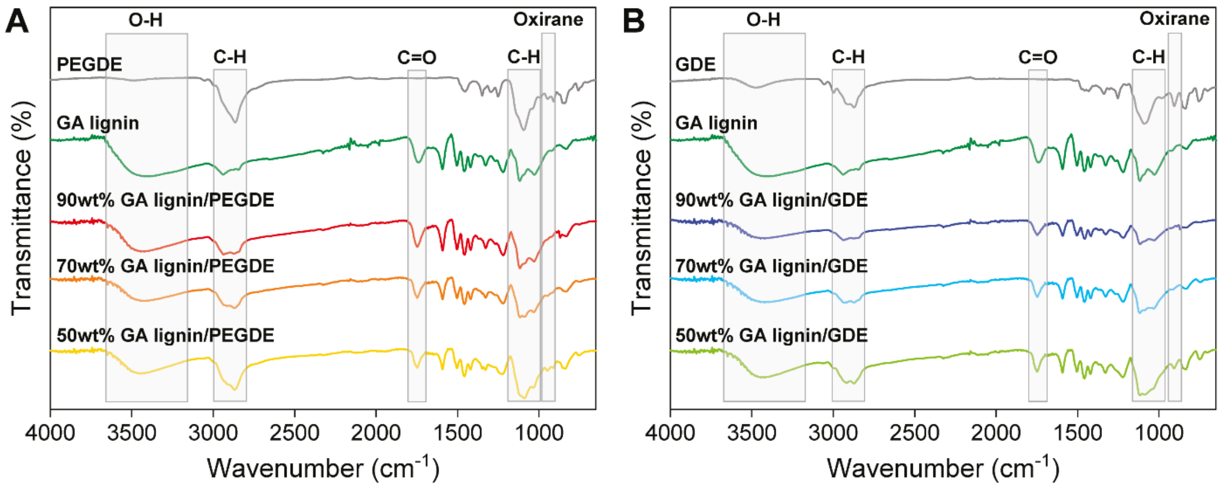

Figure S19. FTIR spectra of **A)** GA lignin/PEGDE and **B)** GA lignin/GDE coatings on fused silica substrates, containing 50, 70 and 90 wt% GA lignin. The spectra of pure PEGDE, GDE and GA lignin are also included for comparison.

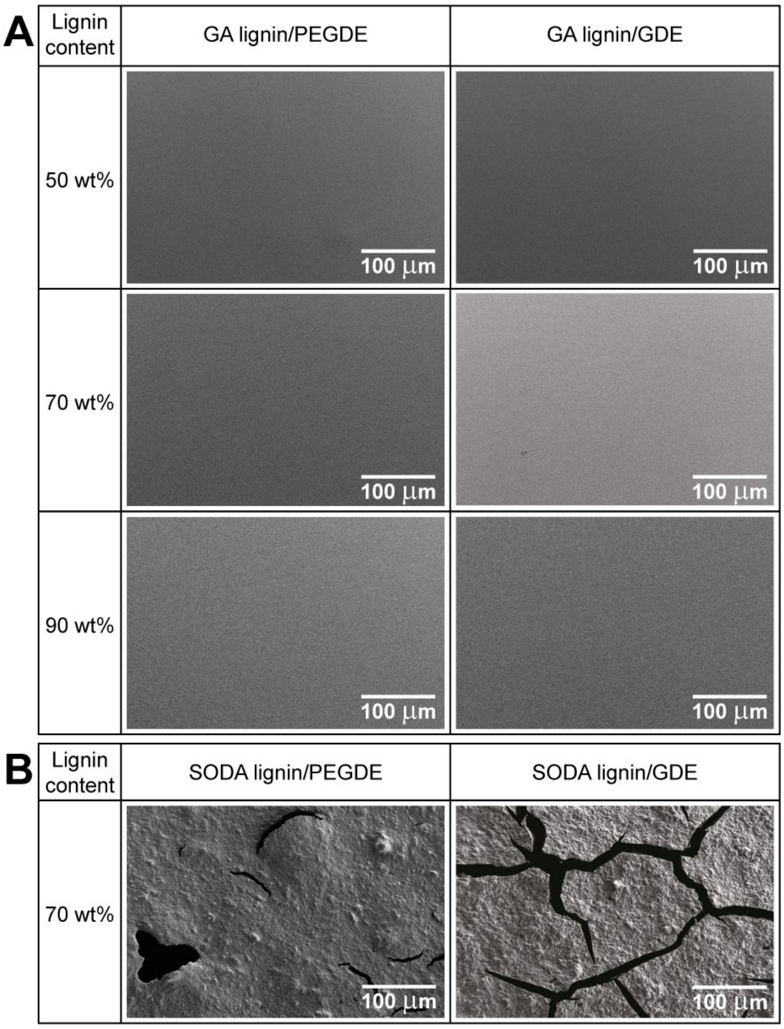

Figure S20. SEM images of **A)** GA lignin coatings prepared by crosslinking with PEGDE and GDE, and **B)** Soda lignin films prepared by crosslinking with PEGDE and GDE.

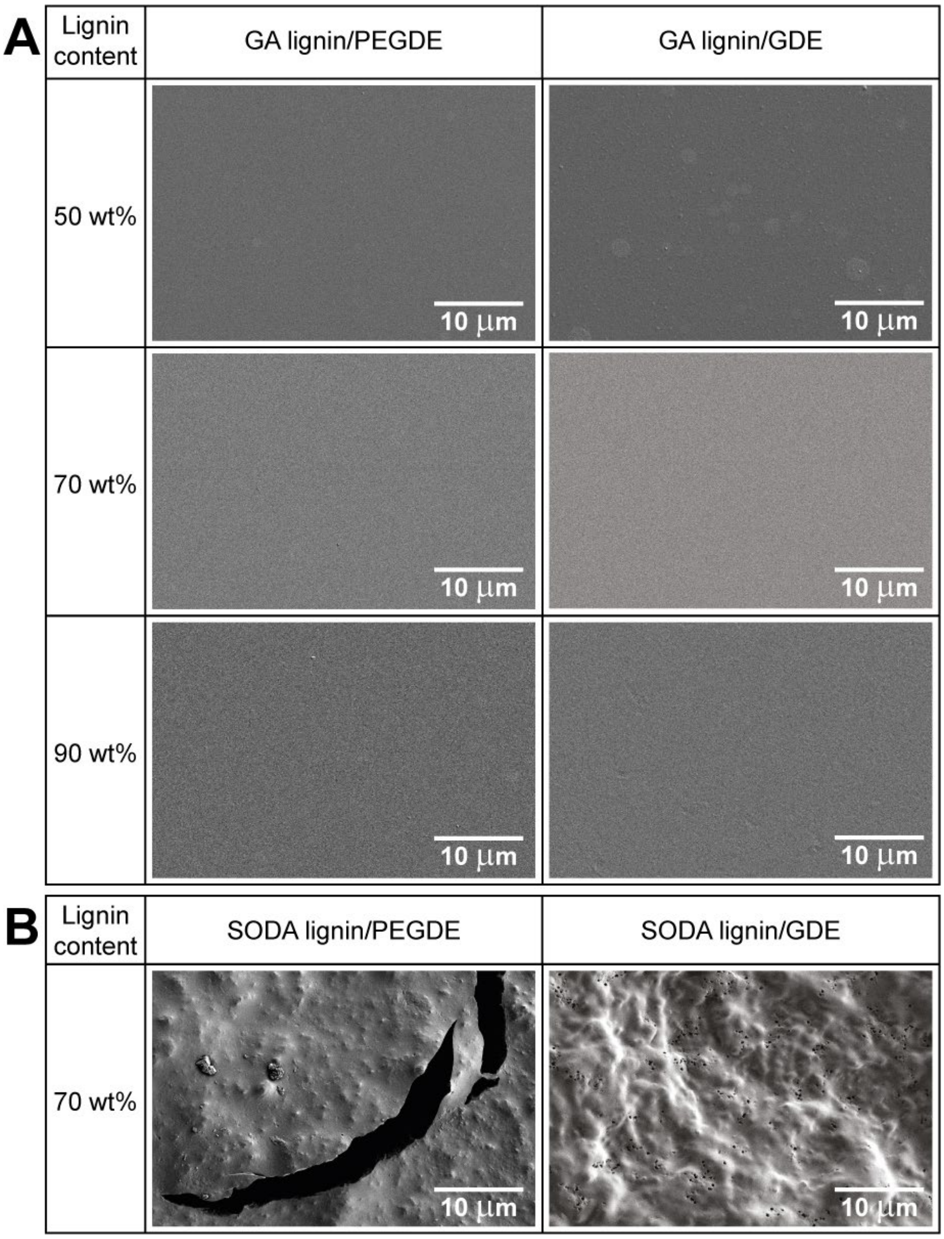

Figure S21. SEM images of **A)** GA lignin films prepared by crosslinking with PEGDE and GDE, and **B)** Soda lignin films prepared by crosslinking with PEGDE and GDE.

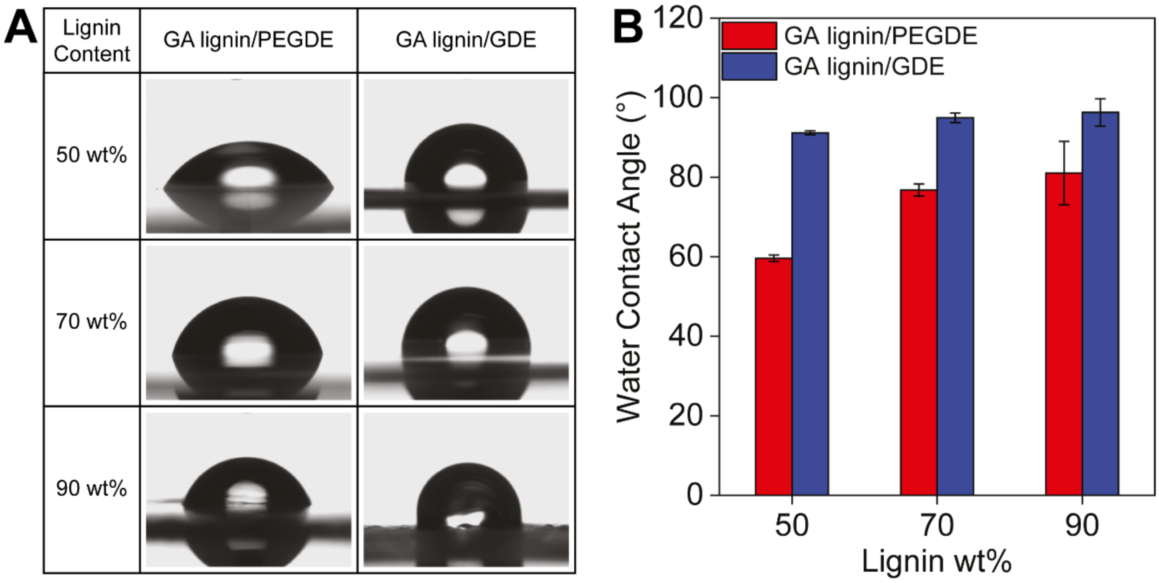

Figure S22. A) Photographs of water droplets deposited onto various GA lignin-based surface coatings. **B)** Water contact angles of GA lignin/PEGDE and GA lignin/GDE surface coatings. Each measurement was repeated three times at different positions on the surface, and the average is reported.

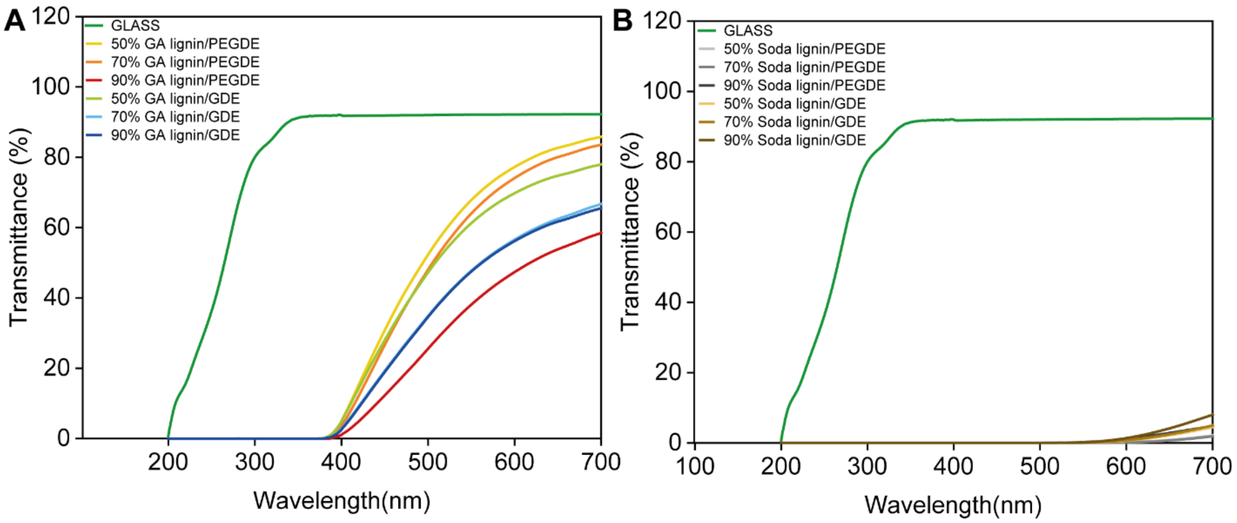

Figure S23. Transmittance (%) spectra recorded from 200 nm to 700 nm covering the visible and the UV range of light through **A)** GA lignin/PEGDE (red) and GA lignin/GDE (blue) coatings on fused silica substrates **B)** Soda-lignin/PEGDE (gray) and Soda-lignin/GDE (brown) coatings on fused silica substrates. A clean glass substrate was measured as control (green).

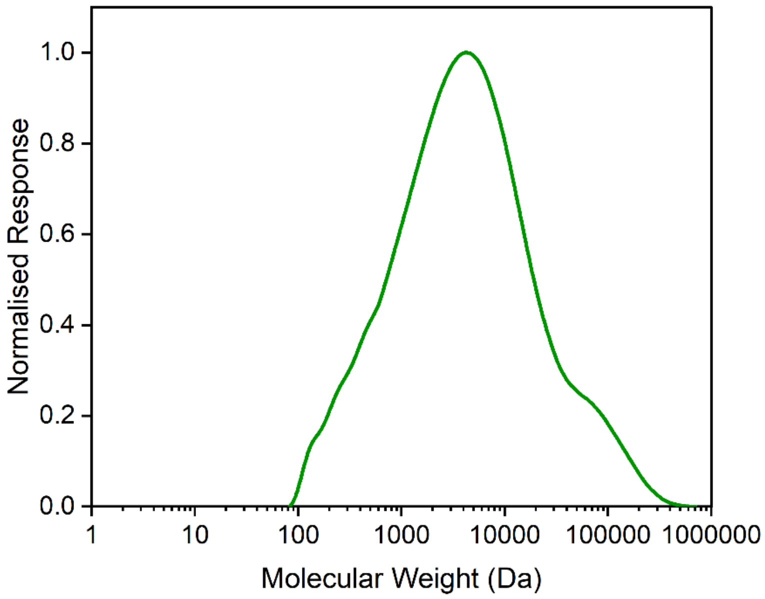

Figure S24. Molecular weight distribution of the GA lignin used in this study.

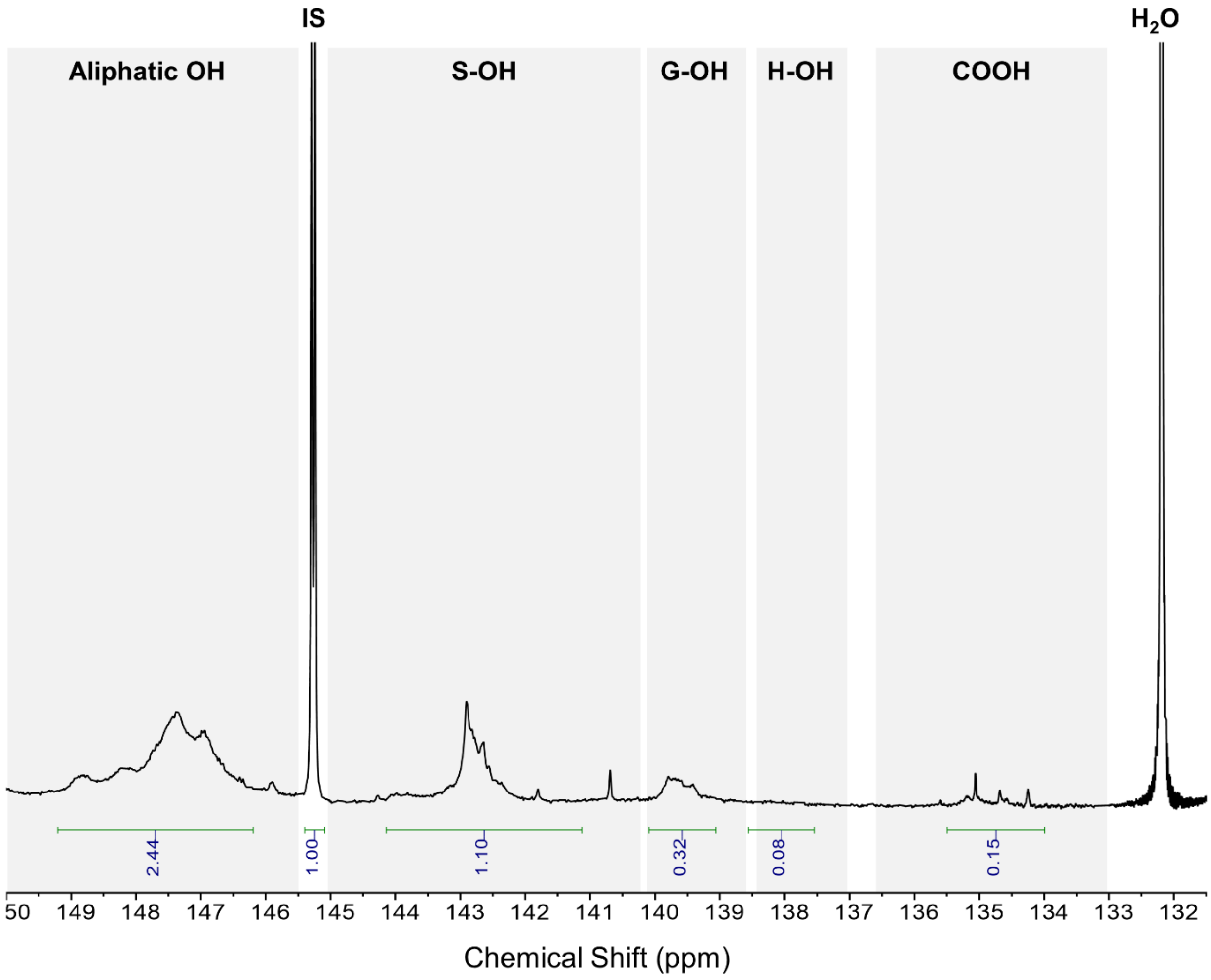

Figure S25. ^{31}P-NMR spectrum of Soda lignin.

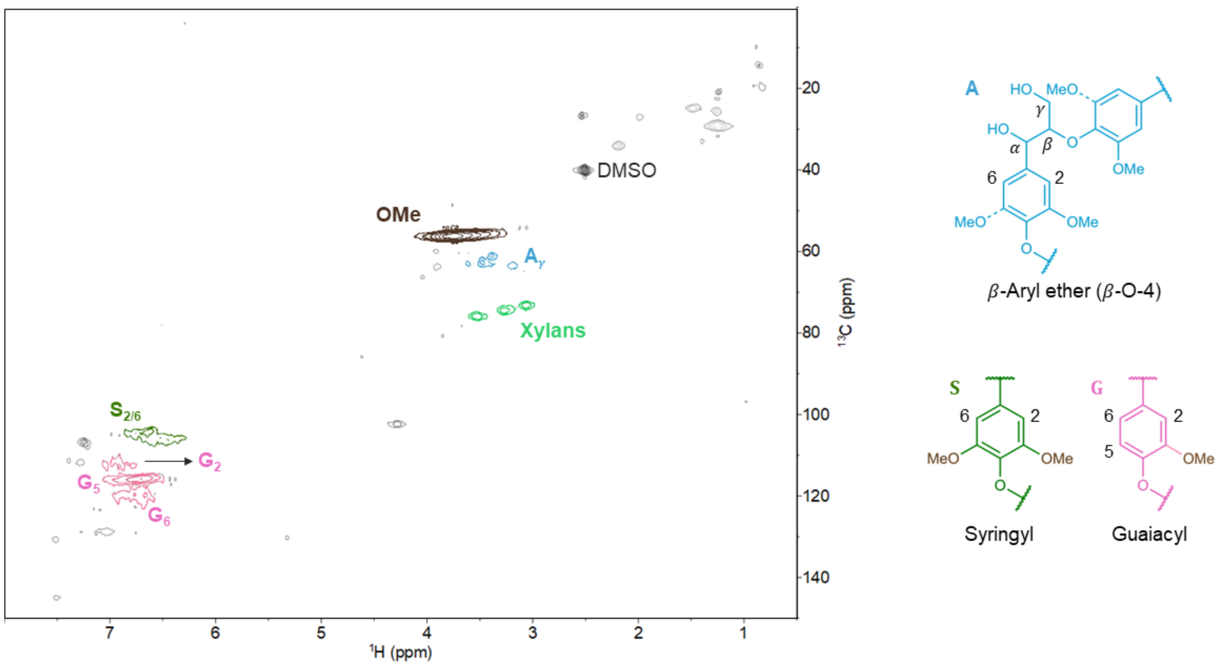

Figure S26. HSQC spectrum of Soda lignin recorded by dissolving 60 mg of sample in 0.5 mL of deuterated DMSO, with the corresponding lignin structures that were assigned to the signals. The unlabeled grey areas correspond to impurities or unresolved signals.

References

(1) Bertella, S.; Bernardes Figueirêdo, M.; De Angelis, G.; Mourez, M.; Bourmaud, C.; Amstad, E.; Luterbacher, J. S. Extraction and Surfactant Properties of Glyoxylic Acid-Functionalized Lignin. *ChemSusChem* **2022**, *15* (15), e202200270. https://doi.org/10.1002/cssc.202200270.

(2) Blois, M. S. Antioxidant Determinations by the Use of a Stable Free Radical. *Nature* **1958**, *181* (4617), 1199–1200. https://doi.org/10.1038/1811199a0.

(3) Bala, R.; Sharma, G. *Digital Color Imaging Handbook*; CRC Press, Boca Raton, 2003.

(4) Handbook of Polymers (Second Edition). In *Handbook of Polymers (Second Edition)*; Wypych, G., Ed.; ChemTec Publishing, 2016; p i. https://doi.org/10.1016/B978-1-895198-92-8.50001-X.

(5) Niemczyk, A.; Dziubek, K.; Sacher-Majewska, B.; Czaja, K.; Czech-Polak, J.; Oliwa, R.; Lenża, J.; Szołyga, M. Thermal Stability and Flame Retardancy of Polypropylene Composites Containing Siloxane-Silsesquioxane Resins. *Polymers* **2018**, *10* (9). https://doi.org/10.3390/polym10091019.

(6) *www.designerdata.nl, Accessed: 10.11.2022.*

(7) Alneamah, M.; Almaamori, M. Study of Thermal Stability of Nitrile Rubber/Polyimide Compounds. *International Journal of Materials and Chemistry* **2015**, *5* (1), 1–3. https://doi.org/10.5923/j.ijmc.20150501.01.

(8) Gordobil, O.; Egüés, I.; Labidi, J. Modification of Eucalyptus and Spruce Organosolv Lignins with Fatty Acids to Use as Filler in PLA. *Reactive and Functional Polymers* **2016**, *104*, 45–52. https://doi.org/10.1016/j.reactfunctpolym.2016.05.002.

(9) Chen, S.-C.; Zhang, X.-M.; Liu, M.; Ma, J.-P.; Lu, W.-Y.; Chen, W.-X. Rheological Characterization and Thermal Stability of Different Intrinsic Viscosity Poly(Ethylene Terephthalate) in Air and Nitrogen. **2016**, *31* (3), 292–300. https://doi.org/10.3139/217.3138.

(10) Jost, V. Packaging Related Properties of Commercially Available Biopolymers – An Overview of the Status Quo. *Express Polymer Letters* **2018**, *12* (5), 429–435. https://doi.org/10.3144/expresspolymlett.2018.36.

(11) Contat-Rodrigo, L.; Ribes-Greus, A.; Imrie, C. T. Thermal Analysis of High-Density Polyethylene and Low-Density Polyethylene with Enhanced Biodegradability. *Journal of Applied Polymer Science* **2002**, *86* (3), 764–772. https://doi.org/10.1002/app.10974.

(12) Zhang, Z.; Cao, H.; Quan, Y.; Ma, R.; Pentzer, E. B.; Green, M. J.; Wang, Q. Thermal Stability and Flammability Studies of MXene–Organic Hybrid Polystyrene Nanocomposites. *Polymers* **2022**, *14* (6). https://doi.org/10.3390/polym14061213.

(13) Abbas-Abadi, M. S.; Van Geem, K. M.; Alvarez, J.; Lopez, G. The Pyrolysis Study of Polybutadiene Rubber under Different Structural and Process Parameters: Comparison with Polyvinyl Chloride Degradation. *Journal of Thermal Analysis and Calorimetry* **2022**, *147* (2), 1237–1249. https://doi.org/10.1007/s10973-020-10431-5.

(14) Liu, S.-H.; Shen, M.-Y.; Kuan, C.-F.; Kuan, H.-C.; Ke, C.-Y.; Chiang, C.-L. Improving Thermal Stability of Polyurethane through the Addition of Hyperbranched Polysiloxane. *Polymers* **2019**, *11* (4). https://doi.org/10.3390/polym11040697.
